# Clinical complexity and hospital admissions in the December holiday period

**DOI:** 10.1371/journal.pone.0234112

**Published:** 2020-06-11

**Authors:** Marco Vincenzo Lenti, Catherine Klersy, Alice Silvia Brera, Valeria Musella, Irene Benedetti, Lucia Padovini, Mariella Ciola, Gabriele Croce, Alessia Ballesio, Maria Fortunata Gorgone, Giampiera Bertolino, Antonio Di Sabatino, Gino Roberto Corazza

**Affiliations:** 1 Department of Internal Medicine, Fondazione IRCCS Policlinico San Matteo, University of Pavia, Pavia, Italy; 2 Biometry and Clinical Epidemiology, Fondazione IRCCS Policlinico San Matteo, University of Pavia, Pavia, Italy; 3 Direzione Medica di Presidio, Fondazione IRCCS Policlinico San Matteo, University of Pavia, Pavia, Italy; Vita Salute University of Milan, ITALY

## Abstract

**Background:**

Christmas and New Year’s holidays are risk factors for hospitalization, but the causes of this “holiday effect” are uncertain. In particular, clinical complexity (CC) has never been assessed in this setting. We therefore sought to determine whether patients admitted to the hospital during the December holiday period had greater CC compared to those admitted during a contiguous non-holiday period.

**Methods:**

This is a prospective, longitudinal study conducted in an academic ward of internal medicine in 2017–2019. Overall, 227 consecutive adult patients were enrolled, including 106 cases (mean age 79.4±12.8 years, 55 females; 15 December-15 January) and 121 controls (mean age 74.3±16.6 years, 56 females; 16 January-16 February). Demographic characteristics, CC, length of stay, and early mortality rate were assessed. Logistic regression analyses for the evaluation of independent correlates of being a holiday case were computed.

**Results:**

Cases displayed greater CC (17.7±5.5 vs 15.2±5.9; p = 0.001), with greater impact of socioeconomic (3.51±1.7 vs 2.9±1.7; p = 0.012) and behavioral (2.36±1.6 vs 1.9±1.8; p = 0.01) CC components. Cases were also significantly frailer according to the Edmonton Frail Scale (8.0±2.8 vs 6.4±3.1; p<0.001), whilst having similar disease burden, as measured by the CIRS comorbidity index. Age (OR 1.02; p = 0.039), low income (OR 1.97, 95% CI 1.10–3.55; p = 0.023), and total CC (OR 1.06; p = 0.014) independently correlated with the cases. Also, cases showed a longer length of stay (median 15.5 *vs* 11 days; p = 0.0016) and higher in-hospital (12 vs 4 events; p = 0.021) and 30-day (14 vs 6 events; p = 0.035) mortality.

**Conclusions:**

Patients hospitalized during the December holiday period had worse health outcomes, and this could be attributable to the grater CC, especially related to socioeconomic (social deprivation, low income) and behavioral factors (inappropriate diet). The evaluation of all CC components could potentially represent a useful tool for a more rational resource allocation over this time of the year.

## Introduction

Christmas and New Year’s holidays are known to be risk factors for hospitalized patients. In fact, previous studies showed that this time of the year is associated with unfavorable health-related outcomes, and this has in turn been ascribed to factors affecting disease burden and/or to reduced staffing levels, work overload and fragmented care [1–4]. However, these factors may only partially explain the observed negative outcomes, as their causative effect has never been demonstrated. Actually, the seasonal “Christmas holiday effect” has also been reported in New Zealand, where the Christmas holiday period falls within the summertime [4]. Further, hospital workload and staff shortage was not found to be associated with excess mortality in patients admitted during weekends and public holidays in the UK [5].

Clinical complexity (CC) is an emerging issue in general internal medicine [5–8], but no information regarding its effect on pattern of hospital admissions over the periods of Christmas and New Year’s is, at present, available. CC is multifaceted and multidimensional, encompassing both biological (age, polypharmacy, multimorbidity, frailty, and level of dependence) and non-biological components (socioeconomic, cultural, behavioral, and environmental) that constantly interact with each other in an unpredictable manner [9–11]. Indeed, coping with CC is one of the most compelling, yet still unmet, needs of modern medicine, given its potential high impact on important health-related outcomes [10–12].

As a part of the ongoing San MAtteo Complexity (SMAC) study, the aim of the present work was to verify whether patients admitted to the hospital over the December holiday period had greater CC, as measured with a mathematical model [9] that has been recently parametrized [13] in the form of a reproducible index [14].

## Material and methods

### Study population

The SMAC study (NCT03439410) is an ongoing research project conducted in an internal medicine unit of an academic hospital (Fondazione IRCCS Policlinico San Matteo, University of Pavia) of northern Italy. Overall, 80 beds are present in the internal medicine unit, and most of the patients are admitted from the local Emergency department. The CC index used in the study (S1 Fig) was developed through a previous consensus meeting [13].

The primary aim of the SMAC study is to evaluate whether the CC index is able to predict patients’ length of stay and the use of healthcare resources. All consecutive adult patients admitted to the ward have been enrolled since November 2017, and patient enrollment ended in November 2019. All patients were enrolled by physicians and healthcare professionals (research nurses) who received specific training before commencing the study [14]. As per protocol design, denial of informed consent and prognosis <24 hours are the only exclusion criteria. In case of cognitive impairment or severe dementia, consent to participate in the study and relevant data were obtained from the caregiver, spouse/partner, a close relative or next of kin, when available. A number of demographic and clinical information was collected, including gender, age, marital status, place of residence, cause of admission to hospital, and number of medications taken upon admission and discharge. In order to compile the CC index, a number of scales are also assessed, including the Cumulative Illness Rating Scale (CIRS) [15], Barthel Index [16], Edmonton Frail Scale [17], and Short Blessed test [18], which reflect disease burden, performance in activities of daily living, frailty, and cognitive disfunction, respectively. In the event of a Short Blessed test score indicative of cognitive impairment (>9), the treating physician had to confirm the diagnosis of dementia.

### Study periods

For the purpose of the present study, only patients admitted between 15 December 2017–15 January 2018 and 15 December 2018–15 January 2019 (cases), and those admitted between 16 January 2018–16 February 2018 and 16 January 2019–16 February 2019 (controls) were included in the analyses, for a total of 64 days per each study group. We selected cases in a holiday period that encompasses both Christmas and New Year’s Eve, during which schools are closed most of the time and public services, especially in peripheral areas, are drastically reduced. We selected a contiguous time of the year for enrollment of the controls, in order to minimize differences between cases and controls in terms of disease epidemiology, especially influenza and respiratory tract infections. Moreover, no statutory holidays fall within that time.

### Outcome assessment

The primary endpoint was to evaluate CC and identify correlates of holiday hospital admissions among CC (total CC index and single CC components) and general demographic characteristics, between cases and controls. The CC was assessed with a CC index that was previously developed involving a multi-professional panel of 25 individuals, including physicians specialized in different areas, general practitioners, nurses, one biostatistician, one patient, and one medical student. In brief, the developmental process of the consensus meeting included the pre-identification of a number of variables capable of qualifying, according to current evidence, each CC domain, and a modified Delphi process through which only five variables per each CC domain were identified [13]. CC components include biological, socioeconomic, cultural, behavioral, and environmental domains (S1 Fig). For the purpose of this study, we scaled each variable of the CC index (S1 Fig) to have a score of 0 if the answer was “no” and a score of 2 if the answer was “yes”; the scores were summed up within each domain (range 0–10) and over all domains (range 0–50). A higher CC index score corresponds to higher complexity. Before administering the CC index, all sub-investigators were trained and became familiar with the use of this tool, as previously reported [14]. Particular attention was given to variables that may be open to different interpretations. For example, by inappropriate diet, we refer to a diet that is likely to be the source of a certain disease or condition, or at least one that contributes to its development. For statistical analysis, the CC index was used as a continuous variable.

As secondary endpoints, we assessed and compared, in cases and controls, the length of stay, early mortality rate (in-hospital and 30-day mortality), and 30-day readmission.

### Statistical analysis

Stata 15 (StataCorp, College Station, TX, USA) was used for all the analyses. The significance level was set at 5% (2-sided). We described continuous variables with the mean and standard deviation or the median and quartiles (IQR), depending on the distribution; we compared them between groups using the Student t test or the Mann Whitney U test. Categorical variables were described as counts and percent and were compared using the Fisher exact test. We used multivariable logistic regression models to identify the independent correlates of holiday hospitalization, reporting odds ratio (OR) and 95% confidence intervals (95% CI) and the area under the ROC curve (AUC-ROC) for model discrimination. We have used three different multivariable regression models, each including the appropriate number of variables. We included non collinear predictors with p<0.1 at multivariable analysis in the models. STROBE reporting guidelines were followed for quality assurance [19]. The protocol was written in accordance with the principles of the Declaration of Helsinki and was approved by the San Matteo Hospital Foundation institutional review board in 2017 (3 July 2017, Protocol number 2017/0019414). All participants provided written informed consent before entering the study. Full statistical analysis, containing all raw data included in the present study, is available as a supporting document. The full dataset of the SMAC study cannot be shared publicly because the research project is still ongoing.

### Sample size calculation

Since this is an exploratory sub-study of the larger SMAC protocol and all “holiday” patients were included, no formal sample size calculation has been performed. However, given that 227 patients were enrolled, of whom 106 were cases, we can compute that 10 binary or continuous covariates could be simultaneously fitted in the proposed logistic multivariable model, according to the 1:10 covariates to events thumb ratio rule to avoid overfitting. Further, considering 106 cases and 121 controls, we were able to show an effect size equal to 0.37 standard deviations (SD) for the primary endpoint. The observed difference was 0.42 SD. According to Cohen [20], considering the CC index, this difference reflects a medium effect size. Finally, the focus of the present study was the evaluation of CC between cases and controls. Hence, no adjustment for multiple endpoints was performed.

## Results

**Fig 1:**
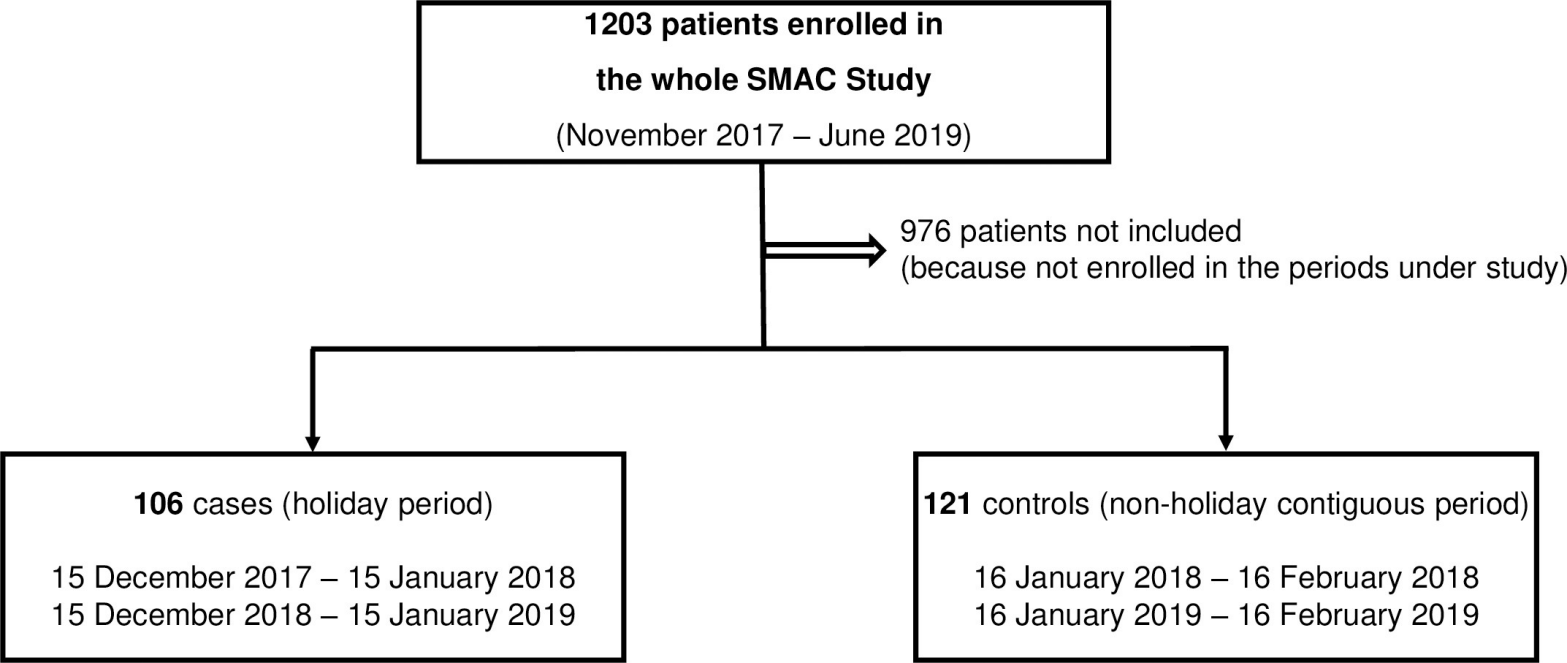
Flowchart of the study. As per protocol designs, two study populations were identified from all patients enrolled in the SMAC study. Patients enrolled in 32 consecutive days during the December holiday period (cases) and 32 consecutive, contiguous days during a non-holiday period (controls) were considered for the purpose of the study. Abbreviations: SMAC, San MAtteo Complexity.

The flowchart of the study is reported in Fig 1. We did not include 976 patients because they were enrolled in a period other than that of the study. Additionally, eight patients did not provide informed consent during the periods of the year under study. Overall, 227 patients were included in the study, of whom 106 were cases and 121 were controls. The global number of physicians and healthcare professionals working in the internal medicine unit during the two periods of the study did not differ. In other words, during holiday periods, compared to a non-holiday period of the year, there is the same number of healthcare professionals working in the ward.

Table 1 reports relevant sociodemographic and clinical baseline characteristics of the two study populations. Notably, cases were significantly older and had a significantly higher total CC score compared to controls. Among CC components, cases had a significantly higher socioeconomic and behavioral CC impact. Cases also had significantly greater frailty, and showed a significantly higher Short Blessed test score compared to controls, whilst having similar disease burden, as measured by the CIRS comorbidity index, polypharmacy and nutritional status. The crude prevalence of patients with dementia was 62/106 (58.5%) for cases and 47/121 (38.8%) for controls (p<0.01). Again, no statistical differences were seen regarding all 14 of the CIRS disease domains and body mass index between the two groups. When taken individually, among all CC index variables, age ≥75 years, frailty, income <1000 €/month, need for a caregiver and inappropriate diet were the only significantly associated variables with cases.

**Table 1:** Sociodemographic and baseline characteristics of 227 patients consecutively admitted to an internal medicine ward during a holiday period (cases; 15 December—15 January) compared to a contiguous period (controls; 16 January—16 February) between 2017–2019.

	Cases	Controls	p
N. of patients (%)	106 (46.7)	121 (53.3)	0.58
Total CCI, mean±SD	17.7±5.5	15.2±5.9	0.001
Gender, female (%)	55 (51.8)	56 (46.2)	0.40
Gender, male (%)	51 (48.2)	65 (53.8)	0.42
Age (years), mean±SD	79.4±12.8	74.3±16.6	0.014
Biological CCI domain, mean±SD	7.8±2.7	7.1±3.1	0.08
Socioeconomic CCI domain, mean±SD	3.51±1.7	2.9±1.7	0.012
Behavioral CCI domain, mean±SD	2.36±1.6	1.9±1.8	0.017
Environmental CCI domain, mean±SD	1.3±1.5	1.0±1.4	0.21
Cultural CCI domain, mean±SD	2.7±2.0	2.2±1.9	0.10
Intake > 5 drugs, n (%)	83 (68.6)	82 (77.3)	0.18
CIRS comorbidity index, mean±SD	3.7±1.5	3.5±1.6	0.23
Short Blessed test, mean±SD	13.0±9.3	10.3±10.4	0.016
Edmonton Frail Scale, mean±SD	8.0±2.8	6.4±3.1	<0.001
Barthel index, mean±SD	79.1±25.5	83.4±21.1	0.26

Abbreviations: CCI, clinical complexity index; CIRS, Cumulative Illness Rating Scale; SD, standard deviation.

Table 2 reports the independent correlates of being a holiday case compared to controls in three different models at multivariable analysis. In Model 1 we included the total CC index, while in Model 2 we only included the components of the CC index variables with p<0.1 at univariable analysis, and in Model 3 we included the CC index domains. Notably, total CC index score, age, income <1000 €/month, and inappropriate diet turned out to be independently correlated with cases. Instead, the need for a caregiver did not turn out to be independently associated with being a holiday case.

**Table 2:** Independent correlates of being a holiday case, including total clinical complexity index (*Model AUC-ROC 1*), single clinical complexity index variables (*Model AUC-ROC 2*), and clinical complexity index domains (*Model AUC-ROC 3*).

	Logistic regression analysis			
	OR	95% CI	p	AUC
*Model AUC-ROC 1*				0.69
Total CCI	1.06	1.01–1.12	0.014	
Age (continuous variable)	1.02	1.00–1.04	0.098	
Gender	1.17	0.68–2.00	0.573	
*Model AUC-ROC 2*				0.69
Edmonton Frail Scale > 5	1.85	0.92–3.72	0.083	
Income < 1000 €/month	1.97	1.10–3.55	0.023	
Home architectural barriers	1.88	0.89–3.95	0.096	
Inappropriate diet	2.29	1.06–4.95	0.036	
Schooling < 8 years	0.93	0.49–1.75	0.818	
Age (continuous variable)	1.02	1.00–1.05	0.039	
Gender	1.07	0.60–1.89	0.817	
*Model AUC-ROC 3*				0.63
Biological CCI	1.07	0.58–1.97	0.827	
Socioeconomic CCI	1.63	0.74–3.59	0.223	
Behavioral CCI	1.93	1.00–3.74	0.051	
Environmental CCI	1.32	0.75–2.31	0.331	
Cultural CCI	1.02	0.57–1.85	0.937	
Age (continuous variable)	1.03	1.01–1.05	0.012	
Gender	1.24	0.72–2.15	0.445	

Abbreviations: AUC, Area Under Curve; CCI, Clinical complexity index; CI, confidence interval; OR, odds ratio.

Table 3 reports the explored clinical outcomes related to the hospital stay in cases and controls. Significantly longer length of stay, higher in-hospital and 30-day mortality were noticed in cases. When adjusting for the CC index, age and sex in a multivariable model, being a holiday case remained significantly associated with increased length of stay (p = 0.001) and in-hospital mortality (p = 0.035), but not with 30-day mortality or readmission (p = 0.185).

**Table 3:** Explored outcomes related to the hospital stay in cases and controls.

	Cases	Controls	p
Length of stay, median days (IQR)	15.5 (11–23)	11 (9–17)	0.001
In-hospital mortality, n (%)	12 (11.3)	4 (3.3)	0.021
30-day mortality, n (%)	14 (13.2)	6 (4.9)	0.035
30-day readmission, n (%)	24 (22.6)	14 (14.1)	0.119

Abbreviation: IQR, interquartile range.

## Discussion

We herein showed that patients admitted to our hospital during the December holiday period display a greater global CC, and in addition are older and with greater frailty as well as more cognitively impaired than those admitted in a contiguous period of the year. Cases and controls did not differ in terms of disease types and burden (as measured by CIRS), while socioeconomic and behavioral CC components were more impaired in holiday cases. Moreover, at logistic regression analysis, total CC index, age, low income, and inappropriate diet were independently correlated with holiday cases. All this translated into a longer length of stay and a higher early mortality.

This study has some weaknesses that should be mentioned. It should be considered as an exploratory, ancillary study, which is part of a broader project on CC that is still ongoing. Generalizability of our results may be limited by the single-center nature of the study, and the relatively small sample size could have underpowered true differences for some parameters. Hence, a replication study, involving different settings, patients and researchers is needed before considering these results transferable and broadly applicable. Nonetheless, we have here investigated in a prospective fashion–and not merely through a registry–a hitherto unexplored area, that is, how CC could affect the pattern of hospital admissions in a particular period of the year. It stands to reason that some negative outcomes could be affected or worsened by a series of factors that our study did not take into account and that could explain the “December holiday effect”, such as hospital overcrowding [21], physicians and nurses understaffing [1], and delays in testing and procedures [22]. Other possible factors, which have never been specifically evaluated, include the point of origin of patients (home vs nursing houses, hospice, etc…), and the different thresholds for seeking care in different periods of the year. All these factors should be the object of future studies. However, what is relevant here is that patients who were admitted to the hospital during the December holiday period differed from those admitted during another time of the year, starting from their baseline characteristics, i.e., since their admission to the hospital. This had been evident for particular patient groups. For example, terminally ill patients may prefer to be home, rather than hospitalized, especially around Christmastime and New Year’s Eve, and this “displacement” hypothesis [1] has been inferred for cancer patients, on the basis of the lower in-hospital mortality found in these patients over this period [2].

Considering our results altogether, it appears clear that contextual, systemic factors are at least not less important than disease-related factors. In fact, socioeconomic and behavioral components of CC–low income, inappropriate diet–seem to interfere more than multimorbidity, disease severity, and polypharmacy in determining significant differences between holiday cases and controls. From a statistical point of view, although the OR for these variables were rather small, the narrow confidence interval is consistent with a high accuracy. Further, from a clinical point of view, low income and poor diet are known to be strictly linked [23], and are both responsible for worse health outcomes, as previously reported [24, 25]. A strict relation between socioeconomic and behavioral components of CC is expected, such as inappropriate diet [26], observed in our patients, as well as the relation between socioeconomic factors and frailty [27].

In our setting, we suggest a “placement” hypothesis, in which the greater CC of hospitalized patients during the December holiday period is mainly related to the high penetrance of detrimental socioeconomic and behavioral factors, in addition to the customary disease severity. Registry studies have already shown that poverty and social deprivation will increase demand on hospital resources by increasing admission and re-admission rates, as well as length of stay [28], particularly over weekends [29]. This in turn leads to higher in-hospital mortality [30] and increased costs burden [31].

The present study highlights the need for reorganizing and implementing healthcare facilities during a stressful time of the year, such as that of the December holiday period. Actually, regardless of the above, the challenging management of more complex patients has never been adequately recognized nor fairly reimbursed, possibly because the few and poor-quality studies published so far have not provided clear evidence that mechanisms for improving their care are cost-effective [32]. A possible explanation is that the concept of “complexity” was linked solely to multimorbidity [8, 9], without considering the socioeconomic and environmental factors. The importance of these latter factors is even more relevant at the territorial level, where better interventions associated with allied healthcare service and enhanced local service may lead to reduction and modification of the pattern of hospital admissions [33]. Hence, the evaluation of all the components of CC could potentially represent a useful tool for more rational resource allocation, and this should be the object of future studies.

## Supporting information

S1 Fig Clinical Complexity (CC) index assessed in the San MAtteo Complexity study. The CC index was developed in a consensus meeting held in 2017, involving 25 panelists with different backgrounds, and can be divided into 5 different components, including biological, socioeconomic, cultural, behavioral, and environmental. Abbreviations: CC, clinical complexity; CIRS, Cumulative Illness Rating Scale.
(PPT)

S1 Data (PDF)

S2 Data (PDF)

## Abbreviations

AUC: area under the curve
CC: clinical complexity
CI: confidence interval
CIRS: Cumulative Illness Rating Scale
DRG: disease related group
IQR: interquartile range
OR: odds ratio
