# Supplementary material for: Clinical complexity and hospital admissions in the December holiday period
Source: PLoS One. 2020 Jun 11;15(6):e0234112. doi: 10.1371/journal.pone.0234112 (PMC7289422; doi:10.1371/journal.pone.0234112)
Supplement: S1 Fig — The CC index was developed in a consensus meeting held in 2017, involving 25 panelists with different backgrounds, and can be divided into 5 different components, including biological, socioeconomic, cultural, behavioral, and environmental. Abbreviations: CC, clinical complexity; CIRS, Cumulative Illness Rating Scale. (PPT) [file pone.0234112.s001.ppt]

## Slide 1
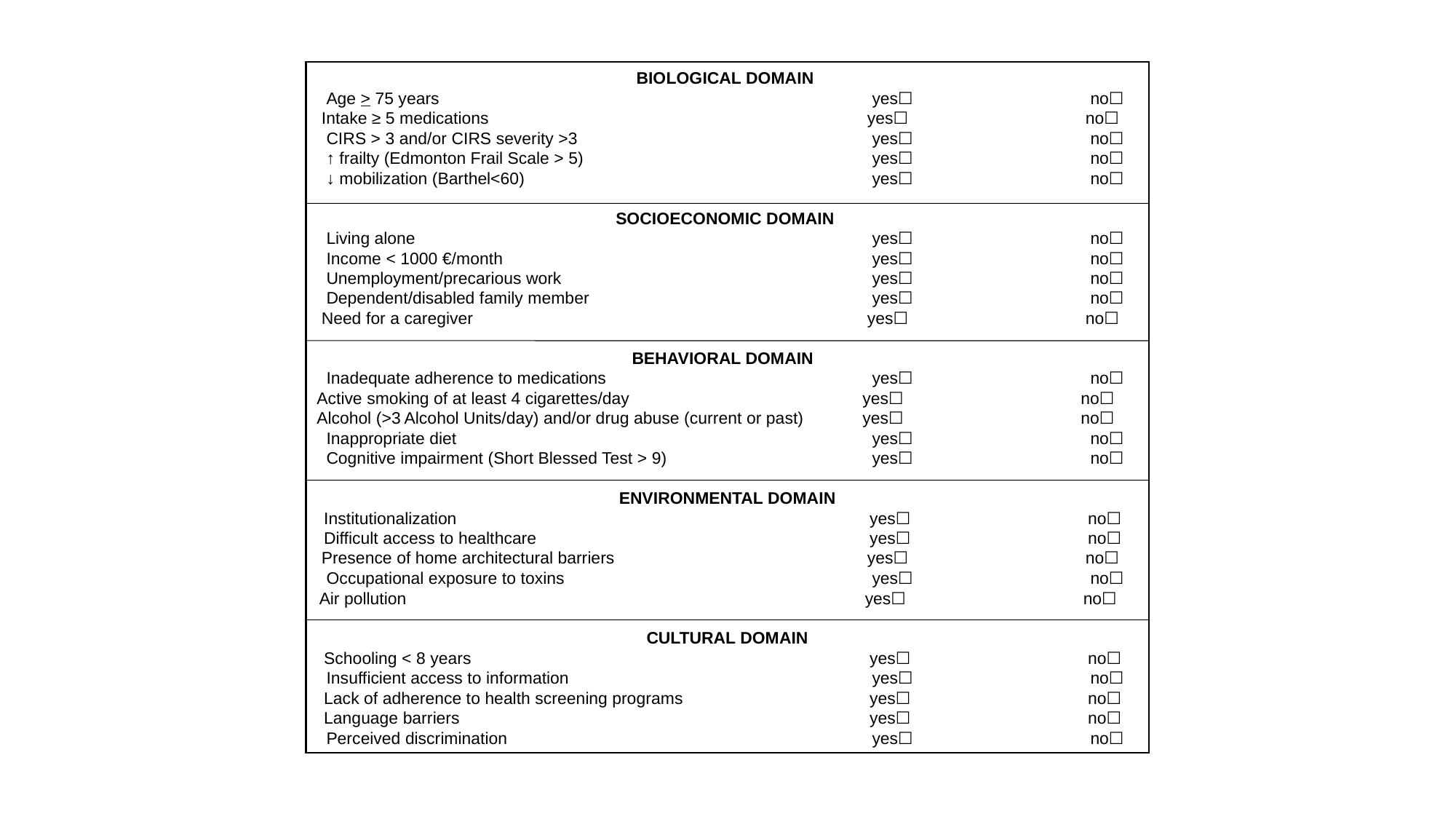

BIOLOGICAL DOMAIN
Age > 75 years				yes☐ 		no☐
Intake ≥ 5 medications				yes☐ 		no☐
CIRS > 3 and/or CIRS severity >3			yes☐ 		no☐
↑ frailty (Edmonton Frail Scale > 5)			yes☐ 		no☐
↓ mobilization (Barthel<60)		 		yes☐ 		no☐
SOCIOECONOMIC DOMAIN
Living alone					yes☐ 		no☐
Income < 1000 €/month				yes☐ 		no☐
Unemployment/precarious work			yes☐ 		no☐
Dependent/disabled family member			yes☐ 		no☐
Need for a caregiver				yes☐ 		no☐
BEHAVIORAL DOMAIN
Inadequate adherence to medications			yes☐ 		no☐
Active smoking of at least 4 cigarettes/day			yes☐ 		no☐
Alcohol (>3 Alcohol Units/day) and/or drug abuse (current or past)	yes☐ 		no☐
Inappropriate diet				yes☐ 		no☐
Cognitive impairment (Short Blessed Test > 9)		yes☐ 		no☐
ENVIRONMENTAL DOMAIN
Institutionalization				yes☐ 		no☐
Difficult access to healthcare				yes☐ 		no☐
Presence of home architectural barriers			yes☐ 		no☐
Occupational exposure to toxins			yes☐ 		no☐
Air pollution					yes☐ 		no☐
CULTURAL DOMAIN
Schooling < 8 years				yes☐ 		no☐
Insufficient access to information			yes☐ 		no☐
Lack of adherence to health screening programs		yes☐ 		no☐
Language barriers				yes☐ 		no☐
Perceived discrimination				yes☐ 		no☐
