# Supplementary material for: Clinical complexity and hospital admissions in the December holiday period
Source: PLoS One. 2020 Jun 11;15(6):e0234112. doi: 10.1371/journal.pone.0234112 (PMC7289422; doi:10.1371/journal.pone.0234112)
Supplement: S1 Data — (PDF) [file pone.0234112.s002.pdf]

```
20 . qui log off

21 .
22 . tab unit

       Unit |      Freq.     Percent        Cum.
------------+-----------------------------------
      MED 1 |        120       50.00       50.00
      MED 2 |        107       44.58       94.58
 Belgioioso |         13        5.42      100.00
------------+-----------------------------------
      Total |        240      100.00

23 . drop if unit=="Belgioioso": unit_ //pagati a giornata
   (13 observations deleted)

24 .
25 . set seed 1234

26 . display in smcl "{newpage}"
```

```
27 .
28 .
29 . *-------------------------------
30 . *(Q1)DESCRIPTIVES BY HOLIDAY CASE
31 . *-------------------------------
32 .
33 . //IMPORTANT NOTE:
34 . //IF MEAN AND MEDIAN CLOSE, USE MEDIAN AND SD + STUDENT T TEST
35 . //IF MEAN AND MEDIAN DIFFERENT, USE MEDIAN AND 25TH-75TH PERCENTILES + MANN WHITNEY U
>  TEST
36 .
37 . tab SELECT
```

SELECT	Freq.	Percent	Cum.
15dec17-15jan18	**63**	**27.75**	**27.75**
16jan18-15feb18	**58**	**25.55**	**53.30**
15dec18-15jan19	**43**	**18.94**	**72.25**
16jan19-15feb19	**63**	**27.75**	**100.00**
Total	**227**	**100.00**	

```
38 . tab CASE
```

HolidayCase	Freq.	Percent	Cum.
regular	**121**	**53.30**	**53.30**
holiday	**106**	**46.70**	**100.00**
Total	**227**	**100.00**	

```
39 .
40 . *1) DEMOGRAPHIC
41 .
42 . local DEMc age bmi

43 .
44 . foreach var of local DEMc       {
  2.         di _newl "*`var' & CASE"
  3.         di _newl
  4.         tabstat `var', by(CASE) c(s)  s(n mea sd min q max) f(%4.2f)
  5.         ttest `var', by(CASE)
  6.         //ranksum `var', by(CASE)
```

```
45 .         di _newl
  7.         }

   *age & CASE


   Summary for variables: age
        by categories of: CASE (HolidayCase)
```

CASE	N	mean	sd	min	p25	p50	p75
regular	**121.00**	**74.39**	**16.67**	**18.00**	**66.00**	**80.00**	**86.00**
holiday	**106.00**	**79.42**	**12.82**	**27.00**	**75.00**	**82.50**	**88.00**
Total	**227.00**	**76.74**	**15.17**	**18.00**	**72.00**	**81.00**	**87.00**

CASE	max
regular	**96.00**
holiday	**98.00**
Total	**98.00**

Two-sample t test with equal variances

Group	Obs	Mean	Std. Err.	Std. Dev.	[95% Conf.	Interval]
regular	**121**	**74.38843**	**1.515002**	**16.66502**	**71.38883**	**77.38803**
holiday	**106**	**79.41509**	**1.245431**	**12.8225**	**76.94563**	**81.88455**
combined	**227**	**76.73568**	**1.006955**	**15.17131**	**74.75146**	**78.7199**
diff		**-5.026665**	**1.994851**		**-8.957645**	**-1.095684**

```
    diff = mean(regular) - mean(holiday)                      t =  -2.5198
Ho: diff = 0                                 degrees of freedom =      225

    Ha: diff < 0                 Ha: diff != 0                 Ha: diff > 0
 Pr(T < t) = 0.0062         Pr(|T| > |t|) = 0.0124          Pr(T > t) = 0.9938
```

***bmi & CASE**

```
   Summary for variables: bmi
        by categories of: CASE (HolidayCase)
```

CASE	N	mean	sd	min	p25	p50	p75
regular	**121.00**	**25.57**	**5.76**	**14.17**	**21.55**	**24.09**	**28.34**
holiday	**106.00**	**25.36**	**5.14**	**15.23**	**21.60**	**24.77**	**29.30**
Total	**227.00**	**25.47**	**5.47**	**14.17**	**21.55**	**24.49**	**28.89**

CASE	max
regular	**53.95**
holiday	**39.06**
Total	**53.95**

Two-sample t test with equal variances

Group	Obs	Mean	Std. Err.	Std. Dev.	[95% Conf.	Interval]
regular	**121**	**25.56983**	**.5236019**	**5.759621**	**24.53314**	**26.60653**
holiday	**106**	**25.35953**	**.4987932**	**5.135391**	**24.37051**	**26.34854**
combined	**227**	**25.47163**	**.3627948**	**5.466055**	**24.75674**	**26.18652**
diff		**.2103065**	**.7286582**		**-1.225561**	**1.646174**

```
    diff = mean(regular) - mean(holiday)                          t =   0.2886
Ho: diff = 0                                     degrees of freedom =      225

    Ha: diff < 0                 Ha: diff != 0                 Ha: diff > 0
 Pr(T < t) = 0.6134         Pr(|T| > |t|) = 0.7731          Pr(T > t) = 0.3866
```

```
.
. local DEM  age_75_years sex BMIclass schooling

.
. foreach var of local DEM       {
  2.         di _newl "*`var' & CASE"
  3.         di _newl
  4.         tab `var' CASE, r co ex
  5.         nptrend `var', by(CASE)
  6.         di _newl
  7.         }

*age_75_years & CASE
```

Key
frequency
row percentage
column percentage

Age >= 75 years	HolidayCase regular	holiday	Total
No	**42**	**23**	**65**
	**64.62**	**35.38**	**100.00**
	**34.71**	**21.70**	**28.63**
Yes	**79**	**83**	**162**
	**48.77**	**51.23**	**100.00**
	**65.29**	**78.30**	**71.37**
Total	**121**	**106**	**227**
	**53.30**	**46.70**	**100.00**
	**100.00**	**100.00**	**100.00**

Fisher's exact = **0.039**
1-sided Fisher's exact = **0.021**

CASE	score	obs	sum of ranks
**regular**	**0**	**121**	**12959.5**
**holiday**	**1**	**106**	**12918.5**

z = **2.16**
Prob > |z| = **0.031**

***sex & CASE**

Key
frequency
row percentage
column percentage

Sex	HolidayCase regular	holiday	Total
M	**65**	**51**	**116**
	**56.03**	**43.97**	**100.00**
	**53.72**	**48.11**	**51.10**
F	**56**	**55**	**111**
	**50.45**	**49.55**	**100.00**
	**46.28**	**51.89**	**48.90**
Total	**121**	**106**	**227**
	**53.30**	**46.70**	**100.00**
	**100.00**	**100.00**	**100.00**

```
         Fisher's exact =                 0.427
 1-sided Fisher's exact =                 0.239
```

CASE	score	obs	sum of ranks
**regular**	**0**	**121**	**13434.5**
**holiday**	**1**	**106**	**12443.5**

```
          z  =  0.84
  Prob > |z| = 0.400
```

***BMIclass & CASE**

Key
frequency
row percentage
column percentage

```
Enumerating sample-space combinations:
stage 4:  enumerations = 1
stage 3:  enumerations = 2
stage 2:  enumerations = 3
stage 1:  enumerations = 0
```

BMIclass	HolidayCase regular	holiday	Total
<18.5	**6**	**6**	**12**
	**50.00**	**50.00**	**100.00**
	**4.96**	**5.66**	**5.29**
[18.5 25[	**61**	**52**	**113**
	**53.98**	**46.02**	**100.00**
	**50.41**	**49.06**	**49.78**
[25 30[	**32**	**27**	**59**
	**54.24**	**45.76**	**100.00**
	**26.45**	**25.47**	**25.99**
>=30	**22**	**21**	**43**
	**51.16**	**48.84**	**100.00**
	**18.18**	**19.81**	**18.94**
Total	**121**	**106**	**227**
	**53.30**	**46.70**	**100.00**
	**100.00**	**100.00**	**100.00**

Fisher's exact = **0.981**

CASE	score	obs	sum of ranks
**regular**	**0**	**121**	**13740**
**holiday**	**1**	**106**	**12138**

z = **0.12**
Prob > |z| = **0.906**

***schooling & CASE**

Key
frequency
row percentage
column percentage

```
Enumerating sample-space combinations:
stage 4:  enumerations = 1
stage 3:  enumerations = 7
stage 2:  enumerations = 58
stage 1:  enumerations = 0
```

Schooling	HolidayCase regular	holiday	Total
< = 5 years	**55**	**59**	**114**
	**48.25**	**51.75**	**100.00**
	**45.45**	**55.66**	**50.22**
6-8 years	**40**	**23**	**63**
	**63.49**	**36.51**	**100.00**
	**33.06**	**21.70**	**27.75**
9-13 years	**19**	**18**	**37**
	**51.35**	**48.65**	**100.00**
	**15.70**	**16.98**	**16.30**
> 13 years	**7**	**6**	**13**
	**53.85**	**46.15**	**100.00**
	**5.79**	**5.66**	**5.73**
Total	**121**	**106**	**227**
	**53.30**	**46.70**	**100.00**
	**100.00**	**100.00**	**100.00**

Fisher's exact = **0.269**

CASE	score	obs	sum of ranks
**regular**	**0**	**121**	**14273.5**
**holiday**	**1**	**106**	**11604.5**

```
         z  = -1.06
Prob > |z| = 0.291
```

```
50 .
51 . display in smcl "{newpage}"
```

```
52 .
53 .
54 . *2) MEDICAL HISTORY
55 .
56 .
57 . local INDc cirs_severity_index cirs_comorbidity_index edmonton_frail_score ///
   >         barthel_index short_blssed_score

58 .
59 . foreach var of local INDc       {
  2.         di _newl "*`var' & CASE"
  3.         di _newl
  4.         tabstat `var', by(CASE) c(s)  s(n mea sd min q max) f(%4.2f)
  5.         //ttest `var', by(CASE)
60 .        ranksum `var', by(CASE)
  6.         di _newl
  7.         }

   *cirs_severity_index & CASE
```

Summary for variables: cirs_severity_index
     by categories of: CASE (HolidayCase)

CASE	N	mean	sd	min	p25	p50	p75
regular	121.00	1.76	0.31	1.15	1.54	1.77	1.92
holiday	106.00	1.80	0.28	1.23	1.62	1.77	2.00
Total	227.00	1.78	0.30	1.15	1.54	1.77	2.00

CASE	max
regular	2.54
holiday	2.38
Total	2.54

Two-sample Wilcoxon rank-sum (Mann-Whitney) test

CASE	obs	rank sum	expected
regular	121	13206.5	13794
holiday	106	12671.5	12084
combined	227	25878	25878

```
unadjusted variance   243694.00
adjustment for ties    -1629.44
                     ----------
adjusted variance     242064.56

Ho: cirs_s~x(CASE==regular) = cirs_s~x(CASE==holiday)
             z =  -1.194
    Prob > |z| =   0.2324
```

***cirs_comorbidity_index & CASE**

Summary for variables: cirs_comorbidity_index
by categories of: CASE (HolidayCase)

CASE	N	mean	sd	min	p25	p50	p75
regular	121.00	3.50	1.62	1.00	2.00	3.00	4.00
holiday	106.00	3.74	1.53	1.00	3.00	4.00	5.00
Total	227.00	3.61	1.58	1.00	2.00	4.00	5.00

CASE	max
regular	8.00
holiday	7.00
Total	8.00

Two-sample Wilcoxon rank-sum (Mann-Whitney) test

CASE	obs	rank sum	expected
regular	121	13211.5	13794
holiday	106	12666.5	12084
combined	227	25878	25878

```
unadjusted variance   243694.00
adjustment for ties    -8147.94
                     ----------
adjusted variance     235546.06

Ho: cirs_c~x(CASE==regular) = cirs_c~x(CASE==holiday)
             z =  -1.200
    Prob > |z| =   0.2301
```

***edmonton_frail_score & CASE**

Summary for variables: edmonton_frail_score
by categories of: CASE (HolidayCase)

CASE	N	mean	sd	min	p25	p50	p75
regular	121.00	6.46	3.17	0.00	4.00	7.00	9.00
holiday	106.00	8.08	2.83	1.00	6.00	8.00	10.00
Total	227.00	7.22	3.11	0.00	5.00	8.00	9.00

CASE	max
regular	13.00
holiday	15.00
Total	15.00

Two-sample Wilcoxon rank-sum (Mann-Whitney) test

CASE	obs	rank sum	expected
regular	121	12095.5	13794
holiday	106	13782.5	12084
combined	227	25878	25878

```
unadjusted variance   243694.00
adjustment for ties    -2716.73
                     ----------
adjusted variance     240977.27

Ho: edmont~e(CASE==regular) = edmont~e(CASE==holiday)
             z =  -3.460
    Prob > |z| =   0.0005
```

***barthel_index & CASE**

Summary for variables: barthel_index
by categories of: CASE (HolidayCase)

CASE	N	mean	sd	min	p25	p50	p75
regular	**121.00**	**83.45**	**21.10**	**18.00**	**71.00**	**95.00**	**100.00**
holiday	**106.00**	**79.11**	**25.52**	**0.00**	**66.00**	**90.50**	**99.00**
Total	**227.00**	**81.42**	**23.32**	**0.00**	**69.00**	**93.00**	**100.00**

CASE	max
regular	**100.00**
holiday	**100.00**
Total	**100.00**

Two-sample Wilcoxon rank-sum (Mann-Whitney) test

CASE	obs	rank sum	expected
regular	**121**	**14336.5**	**13794**
holiday	**106**	**11541.5**	**12084**
combined	**227**	**25878**	**25878**

```
unadjusted variance   243694.00
adjustment for ties     -4722.18
                      ----------
adjusted variance     238971.82

Ho: barthe~x(CASE==regular) = barthe~x(CASE==holiday)
             z =   1.110
    Prob > |z| =   0.2671
```

***short_blssed_score & CASE**

Summary for variables: short_blssed_score
     by categories of: CASE (HolidayCase)

CASE	N	mean	sd	min	p25	p50	p75
regular	**121.00**	**10.38**	**10.45**	**0.00**	**0.00**	**7.00**	**18.00**
holiday	**106.00**	**13.02**	**9.30**	**0.00**	**6.00**	**12.00**	**21.00**
Total	**227.00**	**11.61**	**10.00**	**0.00**	**2.00**	**10.00**	**20.00**

CASE	max
regular	**28.00**
holiday	**28.00**
Total	**28.00**

```
Two-sample Wilcoxon rank-sum (Mann-Whitney) test
```

CASE	obs	rank sum	expected
regular	**121**	**12563.5**	**13794**
holiday	**106**	**13314.5**	**12084**
combined	**227**	**25878**	**25878**

```
unadjusted variance   243694.00
adjustment for ties    -3977.78
                     ----------
adjusted variance     239716.22

Ho: short_~e(CASE==regular) = short_~e(CASE==holiday)
             z =  -2.513
    Prob > |z| =   0.0120
```

```
61 .
62 . local A_DIAG CAPADMicd9cm1 CAPADMicd9cm2 CAPADMicd9cm3

63 .
64 . foreach var of local A_DIAG     {
  2.         di _newl "*`var' & CASE (no test, description only"
  3.         di _newl
  4.         tab `var' CASE, r co
  5.         di _newl
  6.         }

*CAPADMicd9cm1 & CASE (no test, description only
```

Key
frequency
row percentage
column percentage

CAPADMicd9cm1	HolidayCase regular	holiday	Total
1-infett	1	0	1
	100.00	0.00	100.00
	0.83	0.00	0.44
2-tumori	0	1	1
	0.00	100.00	100.00
	0.00	0.94	0.44
3-endocrino	1	7	8
	12.50	87.50	100.00
	0.83	6.60	3.52
4-ematol	10	7	17
	58.82	41.18	100.00
	8.26	6.60	7.49
5-mental	3	0	3
	100.00	0.00	100.00
	2.48	0.00	1.32
6-neurol	4	1	5
	80.00	20.00	100.00
	3.31	0.94	2.20
7-circol	34	28	62
	54.84	45.16	100.00
	28.10	26.42	27.31
8-respir	38	33	71
	53.52	46.48	100.00
	31.40	31.13	31.28
9-digerente	4	6	10
	40.00	60.00	100.00
	3.31	5.66	4.41
10-genitur	2	4	6
	33.33	66.67	100.00
	1.65	3.77	2.64
12-pelle	1	0	1
	100.00	0.00	100.00
	0.83	0.00	0.44
13-osteomusc	2	0	2
	100.00	0.00	100.00
	1.65	0.00	0.88
16-sintomi	15	15	30
	50.00	50.00	100.00

	12.40	14.15	13.22
17-traumi	5	4	9
	55.56	44.44	100.00
	4.13	3.77	3.96
vXX	1	0	1
	100.00	0.00	100.00
	0.83	0.00	0.44
Total	121	106	227
	53.30	46.70	100.00
	100.00	100.00	100.00

***CAPADMicd9cm2 & CASE (no test, description only**

Key
frequency<br>*row percentage*<br>*column percentage*

	HolidayCase		
CAPADMicd9cm2	regular	holiday	Total
1-infett	1	1	2
	50.00	50.00	100.00
	1.89	1.72	1.80
2-tumori	1	1	2
	50.00	50.00	100.00
	1.89	1.72	1.80
3-endocrino	5	6	11
	45.45	54.55	100.00
	9.43	10.34	9.91
4-ematol	1	5	6
	16.67	83.33	100.00
	1.89	8.62	5.41
5-mental	2	0	2
	100.00	0.00	100.00
	3.77	0.00	1.80
6-neurol	1	1	2
	50.00	50.00	100.00

	1.89	1.72	1.80
7-circol	11	17	28
	39.29	60.71	100.00
	20.75	29.31	25.23
8-respir	18	18	36
	50.00	50.00	100.00
	33.96	31.03	32.43
9-digerente	5	1	6
	83.33	16.67	100.00
	9.43	1.72	5.41
10-genitur	4	4	8
	50.00	50.00	100.00
	7.55	6.90	7.21
13-osteomusc	1	0	1
	100.00	0.00	100.00
	1.89	0.00	0.90
16-sintomi	2	4	6
	33.33	66.67	100.00
	3.77	6.90	5.41
17-traumi	1	0	1
	100.00	0.00	100.00
	1.89	0.00	0.90
Total	53	58	111
	47.75	52.25	100.00
	100.00	100.00	100.00

***CAPADMicd9cm3 & CASE (no test, description only**

Key
frequency<br>*row percentage*<br>*column percentage*

CAPADMicd9cm3	HolidayCase regular	holiday	Total
2-tumori	2<br>100.00<br>10.00	0<br>0.00<br>0.00	2<br>100.00<br>5.71
3-endocrino	2<br>100.00<br>10.00	0<br>0.00<br>0.00	2<br>100.00<br>5.71
4-ematol	0<br>0.00<br>0.00	2<br>100.00<br>13.33	2<br>100.00<br>5.71
5-mental	0<br>0.00<br>0.00	1<br>100.00<br>6.67	1<br>100.00<br>2.86
7-circol	4<br>57.14<br>20.00	3<br>42.86<br>20.00	7<br>100.00<br>20.00
8-respir	5<br>62.50<br>25.00	3<br>37.50<br>20.00	8<br>100.00<br>22.86
10-genitur	3<br>60.00<br>15.00	2<br>40.00<br>13.33	5<br>100.00<br>14.29
16-sintomi	3<br>60.00<br>15.00	2<br>40.00<br>13.33	5<br>100.00<br>14.29
17-traumi	1<br>50.00<br>5.00	1<br>50.00<br>6.67	2<br>100.00<br>5.71
vXX	0<br>0.00<br>0.00	1<br>100.00<br>6.67	1<br>100.00<br>2.86
Total	20<br>57.14<br>100.00	15<br>42.86<br>100.00	35<br>100.00<br>100.00

```
65 .
66 .
67 . local CIRS cardiac hypertension vascular respiratory eent upper_gi lower_gi ///
   >         hepatic renal other_gu musculo_skeletal_integum neurological ///
   >         endocrine_metabolic psychiatric_behavioral

68 .
69 . foreach var of local CIRS       {
  2.         di _newl "*`var' & CASE"
  3.         di _newl
  4.         tab `var' CASE, r co ex
  5.         nptrend `var', by(CASE)
  6.         di _newl
  7.         }

*cardiac & CASE
```

Key
frequency
row percentage
column percentage

```
Enumerating sample-space combinations:
stage 5:  enumerations = 1
stage 4:  enumerations = 3
stage 3:  enumerations = 7
stage 2:  enumerations = 38
stage 1:  enumerations = 0
```

1. Cardiac (heart only)	HolidayCase regular	holiday	Total
1	**47**	**33**	**80**
	**58.75**	**41.25**	**100.00**
	**38.84**	**31.13**	**35.24**
2	**3**	**5**	**8**
	**37.50**	**62.50**	**100.00**
	**2.48**	**4.72**	**3.52**
3	**32**	**30**	**62**
	**51.61**	**48.39**	**100.00**
	**26.45**	**28.30**	**27.31**
4	**38**	**37**	**75**
	**50.67**	**49.33**	**100.00**
	**31.40**	**34.91**	**33.04**

5	**1**	**1**	**2**
	**50.00**	**50.00**	**100.00**
	**0.83**	**0.94**	**0.88**
Total	**121**	**106**	**227**
	**53.30**	**46.70**	**100.00**
	**100.00**	**100.00**	**100.00**

Fisher's exact = **0.730**

CASE	score	obs	sum of ranks
**regular**	**0**	**121**	**13351.5**
**holiday**	**1**	**106**	**12526.5**

z = **0.94**
Prob > |z| = **0.345**

***hypertension & CASE**

Key
frequency
row percentage
column percentage

Enumerating sample-space combinations:
stage 4: enumerations = 1
stage 3: enumerations = 2
stage 2: enumerations = 19
stage 1: enumerations = 0

2. Hypertension (rating is based on severity; affected systems are rated separat	HolidayCase regular	holiday	Total
1	**38**	**24**	**62**
	**61.29**	**38.71**	**100.00**
	**31.40**	**22.64**	**27.31**

2	**48**	**49**	**97**
	**49.48**	**50.52**	**100.00**
	**39.67**	**46.23**	**42.73**
3	**34**	**33**	**67**
	**50.75**	**49.25**	**100.00**
	**28.10**	**31.13**	**29.52**
4	**1**	**0**	**1**
	**100.00**	**0.00**	**100.00**
	**0.83**	**0.00**	**0.44**
Total	**121**	**106**	**227**
	**53.30**	**46.70**	**100.00**
	**100.00**	**100.00**	**100.00**

Fisher's exact = **0.343**

CASE	score	obs	sum of ranks
**regular**	**0**	**121**	**13314**
**holiday**	**1**	**106**	**12564**

z = **1.04**
Prob > |z| = **0.299**

***vascular & CASE**

Key
frequency
row percentage
column percentage

```
Enumerating sample-space combinations:
stage 5:  enumerations = 1
stage 4:  enumerations = 2
stage 3:  enumerations = 4
stage 2:  enumerations = 11
stage 1:  enumerations = 0
```

3. Vascular (blood, blood vessels and cells, marrow, spleen, lymphatics)	HolidayCase regular	holiday	Total
1	50<br>52.08<br>41.32	46<br>47.92<br>43.40	96<br>100.00<br>42.29
2	21<br>50.00<br>17.36	21<br>50.00<br>19.81	42<br>100.00<br>18.50
3	40<br>54.79<br>33.06	33<br>45.21<br>31.13	73<br>100.00<br>32.16
4	9<br>60.00<br>7.44	6<br>40.00<br>5.66	15<br>100.00<br>6.61
5	1<br>100.00<br>0.83	0<br>0.00<br>0.00	1<br>100.00<br>0.44
Total	121<br>53.30<br>100.00	106<br>46.70<br>100.00	227<br>100.00<br>100.00

Fisher's exact = **0.936**

CASE	score	obs	sum of ranks
**regular**	0	121	14090.5
**holiday**	1	106	11787.5

z = **-0.64**
Prob > |z| = **0.523**

***respiratory & CASE**

Key
frequency
row percentage
column percentage

```
Enumerating sample-space combinations:
stage 5:  enumerations = 1
stage 4:  enumerations = 3
stage 3:  enumerations = 12
stage 2:  enumerations = 62
stage 1:  enumerations = 0
```

4. Respiratory (lungs, bronchi, trachea below the larynx)	HolidayCase regular	holiday	Total
1	**62**<br>**55.36**<br>**51.24**	**50**<br>**44.64**<br>**47.17**	**112**<br>**100.00**<br>**49.34**
2	**7**<br>**53.85**<br>**5.79**	**6**<br>**46.15**<br>**5.66**	**13**<br>**100.00**<br>**5.73**
3	**18**<br>**42.86**<br>**14.88**	**24**<br>**57.14**<br>**22.64**	**42**<br>**100.00**<br>**18.50**
4	**33**<br>**56.90**<br>**27.27**	**25**<br>**43.10**<br>**23.58**	**58**<br>**100.00**<br>**25.55**
5	**1**<br>**50.00**<br>**0.83**	**1**<br>**50.00**<br>**0.94**	**2**<br>**100.00**<br>**0.88**
Total	**121**<br>**53.30**<br>**100.00**	**106**<br>**46.70**<br>**100.00**	**227**<br>**100.00**<br>**100.00**

Fisher's exact = **0.665**

CASE	score	obs	sum of ranks
**regular**	**0**	**121**	**13684**
**holiday**	**1**	**106**	**12194**

```
          z  =  0.24
  Prob > |z| = 0.810
```

***eent & CASE**

Key
frequency
row percentage
column percentage

```
Enumerating sample-space combinations:
stage 4:  enumerations = 1
stage 3:  enumerations = 2
stage 2:  enumerations = 9
stage 1:  enumerations = 0
```

5. EENT (eye, ear, nose, throat, larynx)	HolidayCase regular	holiday	Total
1	**95**<br>**51.63**<br>**78.51**	**89**<br>**48.37**<br>**83.96**	**184**<br>**100.00**<br>**81.06**
2	**19**<br>**65.52**<br>**15.70**	**10**<br>**34.48**<br>**9.43**	**29**<br>**100.00**<br>**12.78**
3	**6**<br>**46.15**<br>**4.96**	**7**<br>**53.85**<br>**6.60**	**13**<br>**100.00**<br>**5.73**
4	**1**<br>**100.00**<br>**0.83**	**0**<br>**0.00**<br>**0.00**	**1**<br>**100.00**<br>**0.44**
Total	**121**<br>**53.30**<br>**100.00**	**106**<br>**46.70**<br>**100.00**	**227**<br>**100.00**<br>**100.00**

Fisher's exact = **0.358**

CASE	score	obs	sum of ranks
**regular**	**0**	**121**	**14115.5**
**holiday**	**1**	**106**	**11762.5**

```
         z  = -0.95
  Prob > |z| = 0.340
```

***upper_gi & CASE**

Key
frequency
row percentage
column percentage

```
Enumerating sample-space combinations:
stage 5:  enumerations = 1
stage 4:  enumerations = 3
stage 3:  enumerations = 15
stage 2:  enumerations = 90
stage 1:  enumerations = 0
```

6, Upper GI (esophagus, stomach, duodenum, biliary and pancreatic trees; do not	HolidayCase regular	holiday	Total
1	**98**	**76**	**174**
	**56.32**	**43.68**	**100.00**
	**80.99**	**71.70**	**76.65**
2	**15**	**16**	**31**
	**48.39**	**51.61**	**100.00**
	**12.40**	**15.09**	**13.66**
3	**7**	**8**	**15**
	**46.67**	**53.33**	**100.00**
	**5.79**	**7.55**	**6.61**
4	**0**	**5**	**5**
	**0.00**	**100.00**	**100.00**

	**0.00**	**4.72**	**2.20**
5	**1**<br>**50.00**<br>**0.83**	**1**<br>**50.00**<br>**0.94**	**2**<br>**100.00**<br>**0.88**
Total	**121**<br>**53.30**<br>**100.00**	**106**<br>**46.70**<br>**100.00**	**227**<br>**100.00**<br>**100.00**

```
         Fisher's exact =                  0.104
```

CASE	score	obs	sum of ranks
**regular**	**0**	**121**	**13142.5**
**holiday**	**1**	**106**	**12735.5**

```
         z  =  1.78
  Prob > |z| = 0.074
```

***lower_gi & CASE**

Key
frequency<br>*row percentage*<br>*column percentage*

```
Enumerating sample-space combinations:
stage 4:  enumerations = 1
stage 3:  enumerations = 4
stage 2:  enumerations = 31
stage 1:  enumerations = 0
```

7. Lower GI (intestines, hernias)	HolidayCase<br>regular	 holiday	 Total
1	**89**<br>**50.86**<br>**73.55**	**86**<br>**49.14**<br>**81.13**	**175**<br>**100.00**<br>**77.09**
2	**14**<br>**56.00**<br>**11.57**	**11**<br>**44.00**<br>**10.38**	**25**<br>**100.00**<br>**11.01**

3	**14**	**9**	**23**
	**60.87**	**39.13**	**100.00**
	**11.57**	**8.49**	**10.13**
4	**4**	**0**	**4**
	**100.00**	**0.00**	**100.00**
	**3.31**	**0.00**	**1.76**
Total	**121**	**106**	**227**
	**53.30**	**46.70**	**100.00**
	**100.00**	**100.00**	**100.00**

Fisher's exact = **0.237**

CASE	score	obs	sum of ranks
**regular**	**0**	**121**	**14334**
**holiday**	**1**	**106**	**11544**

z = **-1.49**
Prob > |z| = **0.136**

***hepatic & CASE**

Key
frequency
row percentage
column percentage

```
Enumerating sample-space combinations:
stage 4:  enumerations = 1
stage 3:  enumerations = 5
stage 2:  enumerations = 19
stage 1:  enumerations = 0
```

8. Hepatic (liver only)	HolidayCase regular	holiday	Total
1	**106**	**87**	**193**
	**54.92**	**45.08**	**100.00**
	**87.60**	**82.08**	**85.02**
2	**6**	**4**	**10**
	**60.00**	**40.00**	**100.00**
	**4.96**	**3.77**	**4.41**

3	**6**	**10**	**16**
	**37.50**	**62.50**	**100.00**
	**4.96**	**9.43**	**7.05**
4	**3**	**5**	**8**
	**37.50**	**62.50**	**100.00**
	**2.48**	**4.72**	**3.52**
Total	**121**	**106**	**227**
	**53.30**	**46.70**	**100.00**
	**100.00**	**100.00**	**100.00**

Fisher's exact = **0.429**

CASE	score	obs	sum of ranks
**regular**	**0**	**121**	**13412.5**
**holiday**	**1**	**106**	**12465.5**

z = **1.25**
Prob > |z| = **0.213**

***renal & CASE**

Key
frequency
row percentage
column percentage

```
Enumerating sample-space combinations:
stage 4:  enumerations = 1
stage 3:  enumerations = 8
stage 2:  enumerations = 52
stage 1:  enumerations = 0
```

9. Renal (kidneys only)	HolidayCase regular	holiday	Total
1	**89**	**66**	**155**
	**57.42**	**42.58**	**100.00**
	**73.55**	**62.26**	**68.28**
2	**8**	**13**	**21**
	**38.10**	**61.90**	**100.00**
	**6.61**	**12.26**	**9.25**

3	**17**	**17**	**34**
	**50.00**	**50.00**	**100.00**
	**14.05**	**16.04**	**14.98**
4	**7**	**10**	**17**
	**41.18**	**58.82**	**100.00**
	**5.79**	**9.43**	**7.49**
Total	**121**	**106**	**227**
	**53.30**	**46.70**	**100.00**
	**100.00**	**100.00**	**100.00**

Fisher's exact = **0.249**

CASE	score	obs	sum of ranks
**regular**	**0**	**121**	**13092.5**
**holiday**	**1**	**106**	**12785.5**

z = **1.73**
Prob > |z| = **0.084**

***other_gu & CASE**

Key
frequency
row percentage
column percentage

```
Enumerating sample-space combinations:
stage 4:  enumerations = 1
stage 3:  enumerations = 5
stage 2:  enumerations = 26
stage 1:  enumerations = 0
```

10. Other GU (ureters, bladder, urethra, prostate, genitals)	HolidayCase regular	holiday	Total
1	**91**	**90**	**181**
	**50.28**	**49.72**	**100.00**
	**75.21**	**84.91**	**79.74**

2	14<br>66.67<br>11.57	7<br>33.33<br>6.60	21<br>100.00<br>9.25
3	12<br>63.16<br>9.92	7<br>36.84<br>6.60	19<br>100.00<br>8.37
4	4<br>66.67<br>3.31	2<br>33.33<br>1.89	6<br>100.00<br>2.64
Total	121<br>53.30<br>100.00	106<br>46.70<br>100.00	227<br>100.00<br>100.00

Fisher's exact = **0.357**

CASE	score	obs	sum of ranks
**regular**	**0**	**121**	**14411**
**holiday**	**1**	**106**	**11467**

z = **-1.78**
Prob > |z| = **0.075**

***musculo_skeletal_integum & CASE**

Key
frequency<br>*row percentage*<br>*column percentage*

```
Enumerating sample-space combinations:
stage 5:  enumerations = 1
stage 4:  enumerations = 2
stage 3:  enumerations = 8
stage 2:  enumerations = 47
stage 1:  enumerations = 0
```

11. Musculo-skeletal-integumentary (muscles, bone, skin)	HolidayCase regular	holiday	Total
1	**83**	**63**	**146**
	**56.85**	**43.15**	**100.00**
	**68.60**	**59.43**	**64.32**
2	**13**	**18**	**31**
	**41.94**	**58.06**	**100.00**
	**10.74**	**16.98**	**13.66**
3	**21**	**22**	**43**
	**48.84**	**51.16**	**100.00**
	**17.36**	**20.75**	**18.94**
4	**3**	**3**	**6**
	**50.00**	**50.00**	**100.00**
	**2.48**	**2.83**	**2.64**
5	**1**	**0**	**1**
	**100.00**	**0.00**	**100.00**
	**0.83**	**0.00**	**0.44**
Total	**121**	**106**	**227**
	**53.30**	**46.70**	**100.00**
	**100.00**	**100.00**	**100.00**

Fisher's exact = **0.453**

CASE	score	obs	sum of ranks
**regular**	**0**	**121**	**13283**
**holiday**	**1**	**106**	**12595**

z = **1.22**
Prob > |z| = **0.224**

***neurological & CASE**

Key
frequency
row percentage
column percentage

```
Enumerating sample-space combinations:
stage 5:  enumerations = 1
stage 4:  enumerations = 2
stage 3:  enumerations = 7
stage 2:  enumerations = 33
stage 1:  enumerations = 0
```

12. Neurological (brain, spinal cord, nerves; do not include dementia)	HolidayCase regular	holiday	Total
1	87<br>55.06<br>71.90	71<br>44.94<br>66.98	158<br>100.00<br>69.60
2	4<br>80.00<br>3.31	1<br>20.00<br>0.94	5<br>100.00<br>2.20
3	22<br>43.14<br>18.18	29<br>56.86<br>27.36	51<br>100.00<br>22.47
4	7<br>58.33<br>5.79	5<br>41.67<br>4.72	12<br>100.00<br>5.29
5	1<br>100.00<br>0.83	0<br>0.00<br>0.00	1<br>100.00<br>0.44
Total	121<br>53.30<br>100.00	106<br>46.70<br>100.00	227<br>100.00<br>100.00

Fisher's exact = 0.304

CASE	score	obs	sum of ranks
**regular**	**0**	**121**	**13489**
**holiday**	**1**	**106**	**12389**

```
          z  =  0.77
  Prob > |z| = 0.444
```

***endocrine_metabolic & CASE**

Key
frequency
row percentage
column percentage

```
Enumerating sample-space combinations:
stage 5:  enumerations = 1
stage 4:  enumerations = 1
stage 3:  enumerations = 5
stage 2:  enumerations = 20
stage 1:  enumerations = 0
```

13. Endocrine-Metabolic (includes diabetes, diffuse infections, infections, toxi	HolidayCase regular	holiday	Total
1	**25**<br>**50.00**<br>**20.66**	**25**<br>**50.00**<br>**23.58**	**50**<br>**100.00**<br>**22.03**
2	**17**<br>**53.12**<br>**14.05**	**15**<br>**46.88**<br>**14.15**	**32**<br>**100.00**<br>**14.10**
3	**50**<br>**56.82**<br>**41.32**	**38**<br>**43.18**<br>**35.85**	**88**<br>**100.00**<br>**38.77**
4	**28**<br>**50.91**	**27**<br>**49.09**	**55**<br>**100.00**

	23.14	25.47	24.23
5	1<br>50.00<br>0.83	1<br>50.00<br>0.94	2<br>100.00<br>0.88
Total	121<br>53.30<br>100.00	106<br>46.70<br>100.00	227<br>100.00<br>100.00

Fisher's exact = 0.925

CASE	score	obs	sum of ranks
**regular**	0	121	13863.5
**holiday**	1	106	12014.5

z = **-0.15**
Prob > |z| = **0.883**

***psychiatric_behavioral & CASE**

Key
frequency<br>*row percentage*<br>*column percentage*

```
Enumerating sample-space combinations:
stage 4:  enumerations = 1
stage 3:  enumerations = 3
stage 2:  enumerations = 10
stage 1:  enumerations = 0
```

14. Psychiatric/Behavioral (includes depression, anxiety, agitation, psychosis,	HolidayCase<br>regular	 holiday	 Total
1	84<br>55.63<br>69.42	67<br>44.37<br>63.21	151<br>100.00<br>66.52

```
          2 |        11         12 |        23 
            |     47.83      52.17 |    100.00 
            |      9.09      11.32 |     10.13 
------------+----------------------+----------
          3 |        21         21 |        42 
            |     50.00      50.00 |    100.00 
            |     17.36      19.81 |     18.50 
------------+----------------------+----------
          4 |         5          6 |        11 
            |     45.45      54.55 |    100.00 
            |      4.13       5.66 |      4.85 
------------+----------------------+----------
      Total |       121        106 |       227 
            |     53.30      46.70 |    100.00 
            |    100.00     100.00 |    100.00 

           Fisher's exact =                 0.784

           CASE        score          obs   sum of ranks
        regular            0          121        13392.5
        holiday            1          106        12485.5

           z  =  0.97
  Prob > |z| = 0.330


70 . 
71 . local COM cardiac_comorb hypertension_comorb vascular_comorb ///
   >         respiratory_comorb eent_comorb upper_gi_comorb lower_gi_comorb ///
   >         hepatic_comorb renal_comorb musc_skel_integ_comorb other_gu_comorb ///
   >         neurological_comorb endocrine_metabolic_comorb

72 . 
73 . foreach var of local COM        {
  2.         di _newl "*`var' & CASE"
  3.         di _newl
  4.         tab `var' CASE, r co ex
  5.         nptrend `var', by(CASE)
  6.         di _newl
  7.         }

   *cardiac_comorb & CASE
```

Key
frequency
row percentage
column percentage

cardiac comorb	HolidayCase regular	holiday	Total
0	**50**	**38**	**88**
	**56.82**	**43.18**	**100.00**
	**41.32**	**35.85**	**38.77**
1	**71**	**68**	**139**
	**51.08**	**48.92**	**100.00**
	**58.68**	**64.15**	**61.23**
Total	**121**	**106**	**227**
	**53.30**	**46.70**	**100.00**
	**100.00**	**100.00**	**100.00**

Fisher's exact =	**0.416**
1-sided Fisher's exact =	**0.240**

CASE	score	obs	sum of ranks
**regular**	**0**	**121**	**13443**
**holiday**	**1**	**106**	**12435**

z = **0.84**
Prob > |z| = **0.399**

***hypertension_comorb & CASE**

Key
frequency
row percentage
column percentage

hypertension comorb	HolidayCase regular	holiday	Total
0	86	73	159
	54.09	45.91	100.00
	71.07	68.87	70.04
1	35	33	68
	51.47	48.53	100.00
	28.93	31.13	29.96
Total	121	106	227
	53.30	46.70	100.00
	100.00	100.00	100.00

Fisher's exact = **0.772**
1-sided Fisher's exact = **0.414**

CASE	score	obs	sum of ranks
**regular**	**0**	**121**	**13652.5**
**holiday**	**1**	**106**	**12225.5**

z = **0.36**
Prob > |z| = **0.718**

***vascular_comorb & CASE**

Key
frequency
row percentage
column percentage

vascular comorb	HolidayCase regular	holiday	Total
0	71	67	138
	51.45	48.55	100.00
	58.68	63.21	60.79
1	50	39	89
	56.18	43.82	100.00
	41.32	36.79	39.21
Total	121	106	227
	53.30	46.70	100.00
	100.00	100.00	100.00

```
          Fisher's exact =                 0.499
  1-sided Fisher's exact =                 0.288

        CASE        score          obs   sum of ranks
     regular            0          121        14084.5
     holiday            1          106        11793.5

         z  = -0.70
  Prob > |z| = 0.486
```

***respiratory_comorb & CASE**

Key
frequency
row percentage
column percentage

respirator y comorb	HolidayCase regular	holiday	Total
0	69<br>55.20<br>57.02	56<br>44.80<br>52.83	125<br>100.00<br>55.07
1	52<br>50.98<br>42.98	50<br>49.02<br>47.17	102<br>100.00<br>44.93
Total	121<br>53.30<br>100.00	106<br>46.70<br>100.00	227<br>100.00<br>100.00

```
          Fisher's exact =                 0.593
  1-sided Fisher's exact =                 0.308

        CASE        score          obs   sum of ranks
     regular            0          121          13525
     holiday            1          106          12353

         z  =  0.63
  Prob > |z| = 0.527
```

***eent_comorb & CASE**

Key
frequency
row percentage
column percentage

eent comorb	HolidayCase regular	holiday	Total
0	**114**	**99**	**213**
	**53.52**	**46.48**	**100.00**
	**94.21**	**93.40**	**93.83**
1	**7**	**7**	**14**
	**50.00**	**50.00**	**100.00**
	**5.79**	**6.60**	**6.17**
Total	**121**	**106**	**227**
	**53.30**	**46.70**	**100.00**
	**100.00**	**100.00**	**100.00**

Fisher's exact = **1.000**
1-sided Fisher's exact = **0.506**

CASE	score	obs	sum of ranks
**regular**	**0**	**121**	**13741.5**
**holiday**	**1**	**106**	**12136.5**

z = **0.26**
Prob > |z| = **0.799**

***upper_gi_comorb & CASE**

Key
frequency
row percentage
column percentage

upper gi comorb	HolidayCase regular	holiday	Total
0	113	92	205
	55.12	44.88	100.00
	93.39	86.79	90.31
1	8	14	22
	36.36	63.64	100.00
	6.61	13.21	9.69
Total	121	106	227
	53.30	46.70	100.00
	100.00	100.00	100.00

```
        Fisher's exact =                 0.117
1-sided Fisher's exact =                 0.073
```

CASE	score	obs	sum of ranks
**regular**	0	121	13371
**holiday**	1	106	12507

```
         z  =  1.67
Prob > |z| = 0.094
```

***lower_gi_comorb & CASE**

Key
frequency
row percentage
column percentage

lower gi comorb	HolidayCase regular	holiday	Total
0	103	97	200
	51.50	48.50	100.00
	85.12	91.51	88.11
1	18	9	27
	66.67	33.33	100.00
	14.88	8.49	11.89
Total	121	106	227
	53.30	46.70	100.00
	100.00	100.00	100.00

```
          Fisher's exact =                 0.155
  1-sided Fisher's exact =                 0.100

         CASE        score         obs   sum of ranks
      regular            0         121        14203.5
      holiday            1         106        11674.5

         z  = -1.48
  Prob > |z| = 0.139
```

***hepatic_comorb & CASE**

Key
frequency
row percentage
column percentage

hepatic comorb	HolidayCase regular	holiday	Total
0	112<br>55.17<br>92.56	91<br>44.83<br>85.85	203<br>100.00<br>89.43
1	9<br>37.50<br>7.44	15<br>62.50<br>14.15	24<br>100.00<br>10.57
Total	121<br>53.30<br>100.00	106<br>46.70<br>100.00	227<br>100.00<br>100.00

```
          Fisher's exact =                 0.130
  1-sided Fisher's exact =                 0.077

         CASE        score         obs   sum of ranks
      regular            0         121        13363.5
      holiday            1         106        12514.5

         z  =  1.64
  Prob > |z| = 0.102
```

***renal_comorb & CASE**

Key
frequency
row percentage
column percentage

renal comorb	HolidayCase regular	holiday	Total
0	**97**	**79**	**176**
	**55.11**	**44.89**	**100.00**
	**80.17**	**74.53**	**77.53**
1	**24**	**27**	**51**
	**47.06**	**52.94**	**100.00**
	**19.83**	**25.47**	**22.47**
Total	**121**	**106**	**227**
	**53.30**	**46.70**	**100.00**
	**100.00**	**100.00**	**100.00**

Fisher's exact = **0.341**
1-sided Fisher's exact = **0.196**

CASE	score	obs	sum of ranks
**regular**	**0**	**121**	**13432.5**
**holiday**	**1**	**106**	**12445.5**

z = **1.01**
Prob > |z| = **0.311**

***musc_skel_integ_comorb & CASE**

Key
frequency
row percentage
column percentage

musc skel integ comorb	HolidayCase regular	holiday	Total
0	**96**	**81**	**177**
	**54.24**	**45.76**	**100.00**
	**79.34**	**76.42**	**77.97**
1	**25**	**25**	**50**
	**50.00**	**50.00**	**100.00**
	**20.66**	**23.58**	**22.03**
Total	**121**	**106**	**227**
	**53.30**	**46.70**	**100.00**
	**100.00**	**100.00**	**100.00**

Fisher's exact = **0.632**
1-sided Fisher's exact = **0.355**

CASE	score	obs	sum of ranks
**regular**	**0**	121	13606.5
**holiday**	**1**	106	12271.5

z = **0.53**
Prob > |z| = **0.597**

***other_gu_comorb & CASE**

Key
frequency
row percentage
column percentage

other gu comorb	HolidayCase regular	holiday	Total
0	**105**	**97**	**202**
	**51.98**	**48.02**	**100.00**
	**86.78**	**91.51**	**88.99**
1	**16**	**9**	**25**
	**64.00**	**36.00**	**100.00**
	**13.22**	**8.49**	**11.01**
Total	**121**	**106**	**227**
	**53.30**	**46.70**	**100.00**

```
           |    100.00     100.00 |    100.00

          Fisher's exact =                 0.293
  1-sided Fisher's exact =                 0.178

        CASE        score          obs   sum of ranks
     regular            0          121        14097.5
     holiday            1          106        11780.5

        z  = -1.13
  Prob > |z| = 0.257
```

***neurological_comorb & CASE**

Key
frequency
row percentage
column percentage

neurological comorb	HolidayCase regular	holiday	Total
0	**91**	**72**	**163**
	**55.83**	**44.17**	**100.00**
	**75.21**	**67.92**	**71.81**
1	**30**	**34**	**64**
	**46.88**	**53.12**	**100.00**
	**24.79**	**32.08**	**28.19**
Total	**121**	**106**	**227**
	**53.30**	**46.70**	**100.00**
	**100.00**	**100.00**	**100.00**

```
          Fisher's exact =                 0.240
  1-sided Fisher's exact =                 0.143

        CASE        score          obs   sum of ranks
     regular            0          121          13327
     holiday            1          106          12551

        z  =  1.21
  Prob > |z| = 0.225
```

***endocrine_metabolic_comorb & CASE**

Key
frequency
row percentage
column percentage

endocrine metabolic comorb	HolidayCase regular	holiday	Total
0	**42**	**40**	**82**
	**51.22**	**48.78**	**100.00**
	**34.71**	**37.74**	**36.12**
1	**79**	**66**	**145**
	**54.48**	**45.52**	**100.00**
	**65.29**	**62.26**	**63.88**
Total	**121**	**106**	**227**
	**53.30**	**46.70**	**100.00**
	**100.00**	**100.00**	**100.00**

```
          Fisher's exact =                 0.679
  1-sided Fisher's exact =                 0.369
```

CASE	score	obs	sum of ranks
**regular**	**0**	**121**	**13988**
**holiday**	**1**	**106**	**11890**

```
        z  = -0.47
  Prob > |z| = 0.637
```

74 .

```
75 . display in smcl "{newpage}"
```

```
76 .
77 . *3) BASAL VISIT CCI
78 .
79 . local CCIc CCI_TOT

80 .
81 . foreach var of local CCIc       {
  2.         di _newl "*`var' & CASE"
  3.         di _newl
  4.         tabstat `var', by(CASE) c(s)  s(n mea sd min q max) f(%4.2f)
  5.         ttest `var', by(CASE)
  6.         //ranksum `var', by(CASE)
82 .         di _newl
  7.         }

*CCI_TOT & CASE
```

Summary for variables: CCI_TOT
by categories of: CASE (HolidayCase)

CASE	N	mean	sd	min	p25	p50	p75
regular	**121.00**	**15.29**	**5.98**	**0.00**	**10.00**	**16.00**	**20.00**
holiday	**106.00**	**17.74**	**5.54**	**4.00**	**14.00**	**18.00**	**20.00**
Total	**227.00**	**16.43**	**5.89**	**0.00**	**12.00**	**16.00**	**20.00**

CASE	max
regular	**30.00**
holiday	**34.00**
Total	**34.00**

Two-sample t test with equal variances

Group	Obs	Mean	Std. Err.	Std. Dev.	[95% Conf.	Interval]
regular	**121**	**15.28926**	**.5433594**	**5.976953**	**14.21344**	**16.36507**
holiday	**106**	**17.73585**	**.5382581**	**5.541707**	**16.66858**	**18.80312**
combined	**227**	**16.43172**	**.3911642**	**5.893484**	**15.66092**	**17.20251**
diff		**-2.446593**	**.768668**		**-3.961302**	**-.9318838**

```
    diff = mean(regular) - mean(holiday)                          t =  -3.1829
Ho: diff = 0                                     degrees of freedom =      225
```

```
      Ha: diff < 0                 Ha: diff != 0                 Ha: diff > 0
   Pr(T < t) = 0.0008         Pr(|T| > |t|) = 0.0017          Pr(T > t) = 0.9992



83 .
84 .
85 . local CCI CCI_BIO CCI_SOC CCI_BEH CCI_ENV CCI_CUL

86 .
87 . foreach var of local CCI        {
  2.          di _newl "*`var' & CASE"
  3.          di _newl
  4.          tab `var' CASE, r co ex
  5.          di _newl
  6.          tabstat `var', by(CASE) c(s)  s(n mea sd min q max) f(%4.2f)
  7.          //ttest `var', by(CASE)
88 .       ranksum `var', by(CASE)
  8.          di _newl
  9.          }

   *CCI_BIO & CASE


  +-------------------+
  | Key               |
  |-------------------|
  |     frequency     |
  |   row percentage  |
  | column percentage |
  +-------------------+

   Enumerating sample-space combinations:
   stage 7:  enumerations = 1
   stage 6:  enumerations = 7
   stage 5:  enumerations = 56
   stage 4:  enumerations = 214
   stage 3:  enumerations = 1218
   stage 2:  enumerations = 6966
   stage 1:  enumerations = 0
```

CCI_BIO	HolidayCase regular	holiday	Total
0	**6**	**0**	**6**
	**100.00**	**0.00**	**100.00**
	**4.96**	**0.00**	**2.64**
2	**9**	**8**	**17**
	**52.94**	**47.06**	**100.00**
	**7.44**	**7.55**	**7.49**
4	**13**	**11**	**24**
	**54.17**	**45.83**	**100.00**
	**10.74**	**10.38**	**10.57**
6	**23**	**15**	**38**
	**60.53**	**39.47**	**100.00**
	**19.01**	**14.15**	**16.74**
8	**31**	**28**	**59**
	**52.54**	**47.46**	**100.00**
	**25.62**	**26.42**	**25.99**
10	**31**	**35**	**66**
	**46.97**	**53.03**	**100.00**
	**25.62**	**33.02**	**29.07**
12	**8**	**9**	**17**
	**47.06**	**52.94**	**100.00**
	**6.61**	**8.49**	**7.49**
Total	**121**	**106**	**227**
	**53.30**	**46.70**	**100.00**
	**100.00**	**100.00**	**100.00**

Fisher's exact = **0.273**

Summary for variables: CCI_BIO
by categories of: CASE (HolidayCase)

CASE	N	mean	sd	min	p25	p50	p75
regular	**121.00**	**7.12**	**3.12**	**0.00**	**6.00**	**8.00**	**10.00**
holiday	**106.00**	**7.85**	**2.77**	**2.00**	**6.00**	**8.00**	**10.00**
Total	**227.00**	**7.46**	**2.98**	**0.00**	**6.00**	**8.00**	**10.00**

CASE	max
regular	**12.00**
holiday	**12.00**
Total	**12.00**

Two-sample Wilcoxon rank-sum (Mann-Whitney) test

CASE	obs	rank sum	expected
regular	**121**	**12966.5**	**13794**
holiday	**106**	**12911.5**	**12084**
combined	**227**	**25878**	**25878**

```
unadjusted variance   243694.00
adjustment for ties   -11904.34
                      ----------
adjusted variance     231789.66

Ho: CCI_BIO(CASE==regular) = CCI_BIO(CASE==holiday)
             z =  -1.719
    Prob > |z| =   0.0857
```

***CCI_SOC & CASE**

Key
frequency
row percentage
column percentage

```
Enumerating sample-space combinations:
stage 5:  enumerations = 1
stage 4:  enumerations = 4
stage 3:  enumerations = 37
stage 2:  enumerations = 282
stage 1:  enumerations = 0
```

CCI_SOC	HolidayCase regular	holiday	Total
0	17<br>68.00<br>14.05	8<br>32.00<br>7.55	25<br>100.00<br>11.01
2	45<br>59.21<br>37.19	31<br>40.79<br>29.25	76<br>100.00<br>33.48
4	45<br>48.39<br>37.19	48<br>51.61<br>45.28	93<br>100.00<br>40.97
6	13<br>43.33<br>10.74	17<br>56.67<br>16.04	30<br>100.00<br>13.22
8	1<br>33.33<br>0.83	2<br>66.67<br>1.89	3<br>100.00<br>1.32
Total	121<br>53.30<br>100.00	106<br>46.70<br>100.00	227<br>100.00<br>100.00

Fisher's exact = 0.198

Summary for variables: CCI_SOC
by categories of: CASE (HolidayCase)

CASE	N	mean	sd	min	p25	p50	p75
regular	121.00	2.94	1.79	0.00	2.00	2.00	4.00
holiday	106.00	3.51	1.76	0.00	2.00	4.00	4.00
Total	227.00	3.21	1.80	0.00	2.00	4.00	4.00

CASE	max
regular	8.00
holiday	8.00
Total	8.00

Two-sample Wilcoxon rank-sum (Mann-Whitney) test

CASE	obs	rank sum	expected
regular	**121**	**12688**	**13794**
holiday	**106**	**13190**	**12084**
combined	**227**	**25878**	**25878**

```
unadjusted variance    243694.00
adjustment for ties    -26787.67
                      ----------
adjusted variance      216906.33

Ho: CCI_SOC(CASE==regular) = CCI_SOC(CASE==holiday)
             z =  -2.375
    Prob > |z| =   0.0176
```

***CCI_BEH & CASE**

Key
frequency
row percentage
column percentage

```
Enumerating sample-space combinations:
stage 5:  enumerations = 1
stage 4:  enumerations = 5
stage 3:  enumerations = 32
stage 2:  enumerations = 365
stage 1:  enumerations = 0
```

	HolidayCase		
CCI_BEH	regular	holiday	Total
0	**40**	**20**	**60**
	**66.67**	**33.33**	**100.00**
	**33.06**	**18.87**	**26.43**
2	**55**	**53**	**108**
	**50.93**	**49.07**	**100.00**
	**45.45**	**50.00**	**47.58**
4	**20**	**28**	**48**
	**41.67**	**58.33**	**100.00**
	**16.53**	**26.42**	**21.15**
6	**3**	**4**	**7**

	42.86	57.14	100.00
	2.48	3.77	3.08
8	3	1	4
	75.00	25.00	100.00
	2.48	0.94	1.76
Total	121	106	227
	53.30	46.70	100.00
	100.00	100.00	100.00

Fisher's exact = 0.073

Summary for variables: CCI_BEH
by categories of: CASE (HolidayCase)

CASE	N	mean	sd	min	p25	p50	p75
regular	121.00	1.92	1.81	0.00	0.00	2.00	2.00
holiday	106.00	2.36	1.63	0.00	2.00	2.00	4.00
Total	227.00	2.12	1.74	0.00	0.00	2.00	4.00

CASE	max
regular	8.00
holiday	8.00
Total	8.00

Two-sample Wilcoxon rank-sum (Mann-Whitney) test

CASE	obs	rank sum	expected
regular	121	12704	13794
holiday	106	13174	12084
combined	227	25878	25878

unadjusted variance 243694.00
adjustment for ties -33053.04

adjusted variance 210640.96

Ho: CCI_BEH(CASE==regular) = CCI_BEH(CASE==holiday)
z = -2.375
Prob > |z| = 0.0176

***CCI_ENV & CASE**

Key
frequency<br>*row percentage*<br>*column percentage*

Enumerating sample-space combinations:
stage 4: enumerations = 1
stage 3: enumerations = 3
stage 2: enumerations = 18
stage 1: enumerations = 0

CCI_ENV	HolidayCase regular	holiday	Total
0	**71**	**53**	**124**
	**57.26**	**42.74**	**100.00**
	**58.68**	**50.00**	**54.63**
2	**36**	**39**	**75**
	**48.00**	**52.00**	**100.00**
	**29.75**	**36.79**	**33.04**
4	**13**	**11**	**24**
	**54.17**	**45.83**	**100.00**
	**10.74**	**10.38**	**10.57**
6	**1**	**3**	**4**
	**25.00**	**75.00**	**100.00**
	**0.83**	**2.83**	**1.76**
Total	**121**	**106**	**227**
	**53.30**	**46.70**	**100.00**
	**100.00**	**100.00**	**100.00**

Fisher's exact =   **0.424**

Summary for variables: CCI_ENV
     by categories of: CASE (HolidayCase)

CASE	N	mean	sd	min	p25	p50	p75
regular	**121.00**	**1.07**	**1.44**	**0.00**	**0.00**	**0.00**	**2.00**
holiday	**106.00**	**1.32**	**1.56**	**0.00**	**0.00**	**1.00**	**2.00**
Total	**227.00**	**1.19**	**1.50**	**0.00**	**0.00**	**0.00**	**2.00**

CASE	max
regular	**6.00**
holiday	**6.00**
Total	**6.00**

Two-sample Wilcoxon rank-sum (Mann-Whitney) test

CASE	obs	rank sum	expected
regular	**121**	**13244.5**	**13794**
holiday	**106**	**12633.5**	**12084**
combined	**227**	**25878**	**25878**

```
unadjusted variance    243694.00
adjustment for ties    -48796.90
                      ----------
adjusted variance      194897.10

Ho: CCI_ENV(CASE==regular) = CCI_ENV(CASE==holiday)
             z =  -1.245
    Prob > |z| =   0.2132
```

***CCI_CUL & CASE**

Key
frequency
row percentage
column percentage

```
Enumerating sample-space combinations:
stage 5:  enumerations = 1
stage 4:  enumerations = 7
stage 3:  enumerations = 53
stage 2:  enumerations = 561
stage 1:  enumerations = 0
```

CCI_CUL	HolidayCase regular	holiday	Total
0	36	21	57
	63.16	36.84	100.00
	29.75	19.81	25.11
2	46	44	90
	51.11	48.89	100.00
	38.02	41.51	39.65
4	29	30	59
	49.15	50.85	100.00
	23.97	28.30	25.99
6	9	5	14
	64.29	35.71	100.00
	7.44	4.72	6.17
8	1	6	7
	14.29	85.71	100.00
	0.83	5.66	3.08
Total	121	106	227
	53.30	46.70	100.00
	100.00	100.00	100.00

Fisher's exact = 0.105

```
Summary for variables: CCI_CUL
     by categories of: CASE (HolidayCase)
```

CASE	N	mean	sd	min	p25	p50	p75
regular	121.00	2.23	1.90	0.00	0.00	2.00	4.00
holiday	106.00	2.70	2.07	0.00	2.00	2.00	4.00
Total	227.00	2.45	1.99	0.00	0.00	2.00	4.00

CASE	max
regular	**8.00**
holiday	**8.00**
Total	**8.00**

```
Two-sample Wilcoxon rank-sum (Mann-Whitney) test
```

CASE	obs	rank sum	expected
regular	**121**	**13037.5**	**13794**
holiday	**106**	**12840.5**	**12084**
combined	**227**	**25878**	**25878**

```
unadjusted variance   243694.00
adjustment for ties   -23384.91
                      ---------
adjusted variance     220309.09

Ho: CCI_CUL(CASE==regular) = CCI_CUL(CASE==holiday)
             z =  -1.612
    Prob > |z| =   0.1070
```

```
89 .
90 .
91 . local CCI8 CCI_BIO CCI_SOC CCI_BEH CCI_ENV CCI_CUL

92 .
93 . foreach var of local CCI8       {
  2.         di _newl "*`var' (dichotomized at median) & CASE"
  3.         di _newl
  4.         tab M_`var' CASE, r co ex
  5.         di _newl
  6.         }

*CCI_BIO (dichotomized at median) & CASE
```

Key
frequency
row percentage
column percentage

2 quantiles of CCI_BIO	HolidayCase regular	holiday	Total
1	82	62	144
	56.94	43.06	100.00
	67.77	58.49	63.44
2	39	44	83
	46.99	53.01	100.00
	32.23	41.51	36.56
Total	121	106	227
	53.30	46.70	100.00
	100.00	100.00	100.00

Fisher's exact = 0.168
1-sided Fisher's exact = 0.095

***CCI_SOC (dichotomized at median) & CASE**

Key
frequency
row percentage
column percentage

2 quantiles of CCI_SOC	HolidayCase regular	holiday	Total
1	107	87	194
	55.15	44.85	100.00
	88.43	82.08	85.46
2	14	19	33
	42.42	57.58	100.00
	11.57	17.92	14.54
Total	121	106	227
	53.30	46.70	100.00
	100.00	100.00	100.00

Fisher's exact = 0.191
1-sided Fisher's exact = 0.122

***CCI_BEH (dichotomized at median) & CASE**

Key
frequency
row percentage
column percentage

2 quantiles of CCI_BEH	HolidayCase regular	holiday	Total
1	**95**	**73**	**168**
	**56.55**	**43.45**	**100.00**
	**78.51**	**68.87**	**74.01**
2	**26**	**33**	**59**
	**44.07**	**55.93**	**100.00**
	**21.49**	**31.13**	**25.99**
Total	**121**	**106**	**227**
	**53.30**	**46.70**	**100.00**
	**100.00**	**100.00**	**100.00**

Fisher's exact = **0.129**
1-sided Fisher's exact = **0.067**

***CCI_ENV (dichotomized at median) & CASE**

Key
frequency
row percentage
column percentage

2 quantiles of CCI_ENV	HolidayCase regular	holiday	Total
1	**71**	**53**	**124**
	**57.26**	**42.74**	**100.00**
	**58.68**	**50.00**	**54.63**
2	**50**	**53**	**103**
	**48.54**	**51.46**	**100.00**
	**41.32**	**50.00**	**45.37**
Total	**121**	**106**	**227**
	**53.30**	**46.70**	**100.00**
	**100.00**	**100.00**	**100.00**

Fisher's exact = **0.229**
1-sided Fisher's exact = **0.120**

***CCI_CUL (dichotomized at median) & CASE**

Key
frequency
row percentage
column percentage

2 quantiles of CCI_CUL	HolidayCase regular	holiday	Total
1	**82**	**65**	**147**
	**55.78**	**44.22**	**100.00**
	**67.77**	**61.32**	**64.76**
2	**39**	**41**	**80**
	**48.75**	**51.25**	**100.00**
	**32.23**	**38.68**	**35.24**
Total	**121**	**106**	**227**
	**53.30**	**46.70**	**100.00**
	**100.00**	**100.00**	**100.00**

Fisher's exact = **0.332**
1-sided Fisher's exact = **0.191**

```
94 .
95 . display in smcl "{newpage}"
```

```
 96 .
 97 . *3.1) BASAL VISIT CCI SINGLE ITEMS
 98 .
 99 . global BIO age_75_years intake_5_medications cirs_3_or_cirs_severity_3 ///
   >         complete_med_history_form edmonton_frail_scale_5 barthel_index_60

100 .
101 .
102 . global SOC living_alone income_1000_month unemploym_precar_work ///
   >         depend_disab_family_memb need_for_a_caregiver

103 .
104 . global BEH inadeq_adher_to_medicat ///
   >         smoking_at_least_4_cig_day alcohol_or_drug_abuse inappropriate_diet ///
   >         short_blessed_test_9

105 .
106 . global ENV institutionalization difficult_access_to_health ///
   >         home_architect_barriers occupat_expos_to_toxics air_pollution

107 .
108 . global CUL schooling_8_years insuff_access_to_info  ///
   >         lack_adher_screen_programs language_barriers perceived_discrimination

109 .
110 .
111 . foreach glo in global BIO SOC BEH ENV CUL       {
  2.         display as text "{hline 79}"
  3.         foreach var of global `glo'     {
  4.                 di _newl "*`glo': `var' & CASE"
  5.                 di _newl
  6.                 tab `var' CASE, r co ex
  7.                 di _newl
  8.                 di _newl
  9.         }
 10. }
-------------------------------------------------------------------------------
-------------------------------------------------------------------------------

*BIO: age_75_years & CASE


+-------------------+
| Key               |
|-------------------|
|     frequency     |
|  row percentage   |
| column percentage |
+-------------------+
```

Age >= 75 years	HolidayCase regular	holiday	Total
No	**42**	**23**	**65**
	**64.62**	**35.38**	**100.00**
	**34.71**	**21.70**	**28.63**
Yes	**79**	**83**	**162**
	**48.77**	**51.23**	**100.00**
	**65.29**	**78.30**	**71.37**
Total	**121**	**106**	**227**
	**53.30**	**46.70**	**100.00**
	**100.00**	**100.00**	**100.00**

```
          Fisher's exact =                 0.039
  1-sided Fisher's exact =                 0.021
```

***BIO: intake_5_medications & CASE**

Key
frequency
row percentage
column percentage

Intake >= 5 medications	HolidayCase regular	holiday	Total
No	**38**	**24**	**62**
	**61.29**	**38.71**	**100.00**
	**31.40**	**22.64**	**27.31**
Yes	**83**	**82**	**165**
	**50.30**	**49.70**	**100.00**
	**68.60**	**77.36**	**72.69**
Total	**121**	**106**	**227**
	**53.30**	**46.70**	**100.00**
	**100.00**	**100.00**	**100.00**

```
          Fisher's exact =                 0.179
  1-sided Fisher's exact =                 0.092
```

***BIO: cirs_3_or_cirs_severity_3 & CASE**

Key
frequency
row percentage
column percentage

CIRS > 3 and/or CIRS severity > 3	HolidayCase regular	holiday	Total
No	**63**	**49**	**112**
	**56.25**	**43.75**	**100.00**
	**52.07**	**46.67**	**49.56**
Yes	**58**	**56**	**114**
	**50.88**	**49.12**	**100.00**
	**47.93**	**53.33**	**50.44**
Total	**121**	**105**	**226**
	**53.54**	**46.46**	**100.00**
	**100.00**	**100.00**	**100.00**

```
          Fisher's exact =                 0.427
  1-sided Fisher's exact =                 0.249
```

***BIO: complete_med_history_form & CASE**

Key
frequency
row percentage
column percentage

Complete MEDICAL HISTORY FORM	HolidayCase regular	holiday	Total
No	**7**	**4**	**11**
	**63.64**	**36.36**	**100.00**
	**5.83**	**4.35**	**5.19**
Yes	**113**	**88**	**201**
	**56.22**	**43.78**	**100.00**
	**94.17**	**95.65**	**94.81**
Total	**120**	**92**	**212**
	**56.60**	**43.40**	**100.00**
	**100.00**	**100.00**	**100.00**

Fisher's exact = **0.760**
1-sided Fisher's exact = **0.438**

***BIO: edmonton_frail_scale_5 & CASE**

Key
frequency
row percentage
column percentage

Edmonton Frail Scale > 5	HolidayCase regular	holiday	Total
No	42	19	61
	68.85	31.15	100.00
	34.71	17.92	26.87
Yes	79	87	166
	47.59	52.41	100.00
	65.29	82.08	73.13
Total	121	106	227
	53.30	46.70	100.00
	100.00	100.00	100.00

Fisher's exact = 0.005
1-sided Fisher's exact = 0.003

***BIO: barthel_index_60 & CASE**

Key
frequency
row percentage
column percentage

Barthel Index < 60	HolidayCase regular	holiday	Total
No	102	86	188
	54.26	45.74	100.00
	84.30	81.13	82.82
Yes	19	20	39
	48.72	51.28	100.00
	15.70	18.87	17.18
Total	121	106	227
	53.30	46.70	100.00
	100.00	100.00	100.00

Fisher's exact = 0.598
1-sided Fisher's exact = 0.324

---

*SOC: living_alone & CASE

Key
frequency
row percentage
column percentage

Living alone	HolidayCase regular	holiday	Total
No	**87**	**81**	**168**
	**51.79**	**48.21**	**100.00**
	**71.90**	**76.42**	**74.01**
Yes	**34**	**25**	**59**
	**57.63**	**42.37**	**100.00**
	**28.10**	**23.58**	**25.99**
Total	**121**	**106**	**227**
	**53.30**	**46.70**	**100.00**
	**100.00**	**100.00**	**100.00**

Fisher's exact = **0.453**
1-sided Fisher's exact = **0.267**

***SOC: income_1000_month & CASE**

Key
frequency
row percentage
column percentage

Income < 1000€/month	HolidayCase regular	holiday	Total
No	64	38	102
	62.75	37.25	100.00
	52.89	35.85	44.93
Yes	57	68	125
	45.60	54.40	100.00
	47.11	64.15	55.07
Total	121	106	227
	53.30	46.70	100.00
	100.00	100.00	100.00

Fisher's exact = 0.011
1-sided Fisher's exact = 0.007

***SOC: unemploym_precar_work & CASE**

Key
frequency
row percentage
column percentage

Unemployment/precarious work	HolidayCase regular	holiday	Total
No	111	98	209
	53.11	46.89	100.00
	91.74	92.45	92.07
Yes	10	8	18
	55.56	44.44	100.00
	8.26	7.55	7.93
Total	121	106	227
	53.30	46.70	100.00
	100.00	100.00	100.00

Fisher's exact =	**1.000**
1-sided Fisher's exact =	**0.520**

***SOC: depend_disab_family_memb & CASE**

Key
frequency
row percentage
column percentage

Dependent/ disabled family member	HolidayCase regular	holiday	Total
No	**109**<br>**53.17**<br>**90.08**	**96**<br>**46.83**<br>**90.57**	**205**<br>**100.00**<br>**90.31**
Yes	**12**<br>**54.55**<br>**9.92**	**10**<br>**45.45**<br>**9.43**	**22**<br>**100.00**<br>**9.69**
Total	**121**<br>**53.30**<br>**100.00**	**106**<br>**46.70**<br>**100.00**	**227**<br>**100.00**<br>**100.00**

Fisher's exact =	**1.000**
1-sided Fisher's exact =	**0.542**

***SOC: need_for_a_caregiver & CASE**

Key
frequency
row percentage
column percentage

Need for a caregiver	HolidayCase regular	holiday	Total
No	**56**	**31**	**87**
	**64.37**	**35.63**	**100.00**
	**46.28**	**29.25**	**38.33**
Yes	**65**	**75**	**140**
	**46.43**	**53.57**	**100.00**
	**53.72**	**70.75**	**61.67**
Total	**121**	**106**	**227**
	**53.30**	**46.70**	**100.00**
	**100.00**	**100.00**	**100.00**

Fisher's exact = **0.010**
1-sided Fisher's exact = **0.006**

---

*BEH: inadeq_adher_to_medicat & CASE

Key
frequency
row percentage
column percentage

Inadequate adherence to medications	HolidayCase regular	holiday	Total
No	**100**	**88**	**188**
	**53.19**	**46.81**	**100.00**
	**82.64**	**83.81**	**83.19**
Yes	**21**	**17**	**38**
	**55.26**	**44.74**	**100.00**
	**17.36**	**16.19**	**16.81**
Total	**121**	**105**	**226**
	**53.54**	**46.46**	**100.00**
	**100.00**	**100.00**	**100.00**

Fisher's exact = **0.860**
1-sided Fisher's exact = **0.479**

***BEH: smoking_at_least_4_cig_day & CASE**

Key
frequency
row percentage
column percentage

Active smoking of at least 4 cigarettes /day	HolidayCase regular	holiday	Total
No	**103**	**93**	**196**
	**52.55**	**47.45**	**100.00**
	**85.12**	**88.57**	**86.73**
Yes	**18**	**12**	**30**
	**60.00**	**40.00**	**100.00**
	**14.88**	**11.43**	**13.27**
Total	**121**	**105**	**226**
	**53.54**	**46.46**	**100.00**

	100.00	100.00	100.00

Fisher's exact = 0.556
1-sided Fisher's exact = 0.287

***BEH: alcohol_or_drug_abuse & CASE**

Key
frequency
row percentage
column percentage

Alcohol (>3 Unit Alcoholic/ day) and/or drug abuse (current or past)	HolidayCase regular	holiday	Total
No	115	98	213
	53.99	46.01	100.00
	95.04	93.33	94.25
Yes	6	7	13
	46.15	53.85	100.00
	4.96	6.67	5.75
Total	121	105	226
	53.54	46.46	100.00
	100.00	100.00	100.00

Fisher's exact = 0.776
1-sided Fisher's exact = 0.394

***BEH: inappropriate_diet & CASE**

Key
frequency
row percentage
column percentage

Inappropriate diet	HolidayCase regular	holiday	Total
No	**106**	**79**	**185**
	**57.30**	**42.70**	**100.00**
	**87.60**	**75.24**	**81.86**
Yes	**15**	**26**	**41**
	**36.59**	**63.41**	**100.00**
	**12.40**	**24.76**	**18.14**
Total	**121**	**105**	**226**
	**53.54**	**46.46**	**100.00**
	**100.00**	**100.00**	**100.00**

Fisher's exact = **0.024**
1-sided Fisher's exact = **0.013**

***BEH: short_blessed_test_9 & CASE**

Key
frequency
row percentage
column percentage

Short Blessed Test > 9	HolidayCase regular	holiday	Total
No	65	42	107
	60.75	39.25	100.00
	53.72	40.00	47.35
Yes	56	63	119
	47.06	52.94	100.00
	46.28	60.00	52.65
Total	121	105	226
	53.54	46.46	100.00
	100.00	100.00	100.00

```
         Fisher's exact =                 0.046
 1-sided Fisher's exact =                 0.027
```

---

*ENV: institutionalization & CASE

Key
frequency
row percentage
column percentage

Institutionalization	HolidayCase regular	holiday	Total
No	116	98	214
	54.21	45.79	100.00
	95.87	92.45	94.27
Yes	5	8	13
	38.46	61.54	100.00
	4.13	7.55	5.73
Total	121	106	227
	53.30	46.70	100.00
	100.00	100.00	100.00

Fisher's exact = 0.392
1-sided Fisher's exact = 0.207

***ENV: difficult_access_to_health & CASE**

Key
frequency
row percentage
column percentage

Difficult access to healthcare	HolidayCase regular	holiday	Total
No	**107**	**94**	**201**
	**53.23**	**46.77**	**100.00**
	**88.43**	**88.68**	**88.55**
Yes	**14**	**12**	**26**
	**53.85**	**46.15**	**100.00**
	**11.57**	**11.32**	**11.45**
Total	**121**	**106**	**227**
	**53.30**	**46.70**	**100.00**
	**100.00**	**100.00**	**100.00**

Fisher's exact = 1.000
1-sided Fisher's exact = 0.561

***ENV: home_architect_barriers & CASE**

Key
frequency
row percentage
column percentage

Presence of home architectural barriers	HolidayCase regular	holiday	Total
No	**106**	**82**	**188**
	**56.38**	**43.62**	**100.00**
	**87.60**	**77.36**	**82.82**
Yes	**15**	**24**	**39**
	**38.46**	**61.54**	**100.00**
	**12.40**	**22.64**	**17.18**
Total	**121**	**106**	**227**
	**53.30**	**46.70**	**100.00**
	**100.00**	**100.00**	**100.00**

Fisher's exact = **0.052**
1-sided Fisher's exact = **0.031**

***ENV: occupat_expos_to_toxics & CASE**

Key
frequency
row percentage
column percentage

Occupational exposure to toxics	HolidayCase regular	holiday	Total
No	109	90	199
	54.77	45.23	100.00
	90.08	84.91	87.67
Yes	12	16	28
	42.86	57.14	100.00
	9.92	15.09	12.33
Total	121	106	227
	53.30	46.70	100.00
	100.00	100.00	100.00

Fisher's exact = 0.312
1-sided Fisher's exact = 0.163

***ENV: air_pollution & CASE**

Key
frequency
row percentage
column percentage

Air pollution	HolidayCase regular	holiday	Total
No	102	96	198
	51.52	48.48	100.00
	84.30	90.57	87.22
Yes	19	10	29
	65.52	34.48	100.00
	15.70	9.43	12.78
Total	121	106	227
	53.30	46.70	100.00
	100.00	100.00	100.00

```
         Fisher's exact =                 0.170
 1-sided Fisher's exact =                 0.112
```

---

*CUL: schooling_8_years & CASE

Key
frequency
row percentage
column percentage

Schooling < 8 years	HolidayCase regular	holiday	Total
No	**64**	**43**	**107**
	**59.81**	**40.19**	**100.00**
	**52.89**	**40.57**	**47.14**
Yes	**57**	**63**	**120**
	**47.50**	**52.50**	**100.00**
	**47.11**	**59.43**	**52.86**
Total	**121**	**106**	**227**
	**53.30**	**46.70**	**100.00**
	**100.00**	**100.00**	**100.00**

```
         Fisher's exact =                 0.083
 1-sided Fisher's exact =                 0.042
```

***CUL: insuff_access_to_info & CASE**

Key
frequency<br>*row percentage*<br>*column percentage*

Insufficient access to information	HolidayCase regular	holiday	Total
No	**82**<br>**54.30**<br>**67.77**	**69**<br>**45.70**<br>**65.09**	**151**<br>**100.00**<br>**66.52**
Yes	**39**<br>**51.32**<br>**32.23**	**37**<br>**48.68**<br>**34.91**	**76**<br>**100.00**<br>**33.48**
Total	**121**<br>**53.30**<br>**100.00**	**106**<br>**46.70**<br>**100.00**	**227**<br>**100.00**<br>**100.00**

Fisher's exact = **0.675**
1-sided Fisher's exact = **0.388**

***CUL: lack_adher_screen_programs & CASE**

Key
frequency<br>*row percentage*<br>*column percentage*

Lack of adherence to health screening programs	HolidayCase regular	holiday	Total
No	88<br>54.32<br>72.73	74<br>45.68<br>69.81	162<br>100.00<br>71.37
Yes	33<br>50.77<br>27.27	32<br>49.23<br>30.19	65<br>100.00<br>28.63
Total	121<br>53.30<br>100.00	106<br>46.70<br>100.00	227<br>100.00<br>100.00

Fisher's exact = **0.661**
1-sided Fisher's exact = **0.367**

***CUL: language_barriers & CASE**

Key
frequency<br>*row percentage*<br>*column percentage*

Language barriers	HolidayCase regular	holiday	Total
No	117<br>53.92<br>96.69	100<br>46.08<br>94.34	217<br>100.00<br>95.59
Yes	4<br>40.00<br>3.31	6<br>60.00<br>5.66	10<br>100.00<br>4.41
Total	121<br>53.30<br>100.00	106<br>46.70<br>100.00	227<br>100.00<br>100.00

```
         Fisher's exact =                 0.521
 1-sided Fisher's exact =                 0.295
```

***CUL: perceived_discrimination & CASE**

Key
frequency
row percentage
column percentage

Perceived discrimination	HolidayCase regular	holiday	Total
No	**119**	**101**	**220**
	**54.09**	**45.91**	**100.00**
	**98.35**	**95.28**	**96.92**
Yes	**2**	**5**	**7**
	**28.57**	**71.43**	**100.00**
	**1.65**	**4.72**	**3.08**
Total	**121**	**106**	**227**
	**53.30**	**46.70**	**100.00**
	**100.00**	**100.00**	**100.00**

```
         Fisher's exact =                 0.256
 1-sided Fisher's exact =                 0.172
```

```
112 . display in smcl "{newpage}"
```

```
113 .
114 .
115 . *DISCHARGE DIAGNOSIS, TREATMENT AND DRG
116 .
117 .
118 . local DIS CAPDISCHicd9cm1 DRG

119 .
120 . foreach var of local DIS        {
  2.         di _newl "*`var' & CASE (no test, description only"
  3.         di _newl
  4.         tab `var' CASE, r co
  5.         di _newl
  6.         }

*CAPDISCHicd9cm1 & CASE (no test, description only
```

Key
frequency
row percentage
column percentage

CAPDISCHicd9c m1	HolidayCase regular	holiday	Total
1-infett	**2**	**6**	**8**
	**25.00**	**75.00**	**100.00**
	**1.67**	**5.66**	**3.54**
2-tumori	**6**	**6**	**12**
	**50.00**	**50.00**	**100.00**
	**5.00**	**5.66**	**5.31**
3-endocrino	**5**	**5**	**10**
	**50.00**	**50.00**	**100.00**
	**4.17**	**4.72**	**4.42**
4-ematol	**8**	**7**	**15**
	**53.33**	**46.67**	**100.00**
	**6.67**	**6.60**	**6.64**
5-mental	**1**	**1**	**2**
	**50.00**	**50.00**	**100.00**
	**0.83**	**0.94**	**0.88**
6-neurol	**5**	**1**	**6**

	83.33	16.67	100.00
	4.17	0.94	2.65
7-circol	34	31	65
	52.31	47.69	100.00
	28.33	29.25	28.76
8-respir	38	33	71
	53.52	46.48	100.00
	31.67	31.13	31.42
9-digerente	11	3	14
	78.57	21.43	100.00
	9.17	2.83	6.19
10-genitur	4	7	11
	36.36	63.64	100.00
	3.33	6.60	4.87
13-osteomusc	1	0	1
	100.00	0.00	100.00
	0.83	0.00	0.44
16-sintomi	3	4	7
	42.86	57.14	100.00
	2.50	3.77	3.10
17-traumi	2	2	4
	50.00	50.00	100.00
	1.67	1.89	1.77
Total	120	106	226
	53.10	46.90	100.00
	100.00	100.00	100.00

***DRG & CASE (no test, description only**

Key
frequency<br>*row percentage*<br>*column percentage*

DRG	HolidayCase regular	holiday	Total
12	**2**<br>**100.00**<br>**1.68**	**0**<br>**0.00**<br>**0.00**	**2**<br>**100.00**<br>**0.89**
14	**1**<br>**33.33**<br>**0.84**	**2**<br>**66.67**<br>**1.89**	**3**<br>**100.00**<br>**1.33**
68	**2**<br>**100.00**<br>**1.68**	**0**<br>**0.00**<br>**0.00**	**2**<br>**100.00**<br>**0.89**
76	**0**<br>**0.00**<br>**0.00**	**1**<br>**100.00**<br>**0.94**	**1**<br>**100.00**<br>**0.44**
78	**4**<br>**50.00**<br>**3.36**	**4**<br>**50.00**<br>**3.77**	**8**<br>**100.00**<br>**3.56**
79	**1**<br>**50.00**<br>**0.84**	**1**<br>**50.00**<br>**0.94**	**2**<br>**100.00**<br>**0.89**
80	**2**<br>**100.00**<br>**1.68**	**0**<br>**0.00**<br>**0.00**	**2**<br>**100.00**<br>**0.89**
82	**1**<br>**100.00**<br>**0.84**	**0**<br>**0.00**<br>**0.00**	**1**<br>**100.00**<br>**0.44**
85	**0**<br>**0.00**<br>**0.00**	**1**<br>**100.00**<br>**0.94**	**1**<br>**100.00**<br>**0.44**
87	**6**<br>**66.67**<br>**5.04**	**3**<br>**33.33**<br>**2.83**	**9**<br>**100.00**<br>**4.00**
88	**7**<br>**87.50**<br>**5.88**	**1**<br>**12.50**<br>**0.94**	**8**<br>**100.00**<br>**3.56**
89	**20**<br>**50.00**<br>**16.81**	**20**<br>**50.00**<br>**18.87**	**40**<br>**100.00**<br>**17.78**
90	**0**<br>**0.00**	**1**<br>**100.00**	**1**<br>**100.00**

	0.00	0.94	0.44
99	1<br>100.00<br>0.84	0<br>0.00<br>0.00	1<br>100.00<br>0.44
110	0<br>0.00<br>0.00	1<br>100.00<br>0.94	1<br>100.00<br>0.44
121	8<br>80.00<br>6.72	2<br>20.00<br>1.89	10<br>100.00<br>4.44
122	1<br>50.00<br>0.84	1<br>50.00<br>0.94	2<br>100.00<br>0.89
123	0<br>0.00<br>0.00	2<br>100.00<br>1.89	2<br>100.00<br>0.89
124	1<br>100.00<br>0.84	0<br>0.00<br>0.00	1<br>100.00<br>0.44
127	17<br>44.74<br>14.29	21<br>55.26<br>19.81	38<br>100.00<br>16.89
131	2<br>100.00<br>1.68	0<br>0.00<br>0.00	2<br>100.00<br>0.89
138	1<br>33.33<br>0.84	2<br>66.67<br>1.89	3<br>100.00<br>1.33
140	0<br>0.00<br>0.00	1<br>100.00<br>0.94	1<br>100.00<br>0.44
141	0<br>0.00<br>0.00	1<br>100.00<br>0.94	1<br>100.00<br>0.44
144	1<br>100.00<br>0.84	0<br>0.00<br>0.00	1<br>100.00<br>0.44
157	1<br>100.00<br>0.84	0<br>0.00<br>0.00	1<br>100.00<br>0.44

172	1<br>50.00<br>0.84	1<br>50.00<br>0.94	2<br>100.00<br>0.89
174	2<br>66.67<br>1.68	1<br>33.33<br>0.94	3<br>100.00<br>1.33
179	5<br>83.33<br>4.20	1<br>16.67<br>0.94	6<br>100.00<br>2.67
182	0<br>0.00<br>0.00	1<br>100.00<br>0.94	1<br>100.00<br>0.44
183	0<br>0.00<br>0.00	1<br>100.00<br>0.94	1<br>100.00<br>0.44
188	1<br>100.00<br>0.84	0<br>0.00<br>0.00	1<br>100.00<br>0.44
191	0<br>0.00<br>0.00	1<br>100.00<br>0.94	1<br>100.00<br>0.44
201	1<br>100.00<br>0.84	0<br>0.00<br>0.00	1<br>100.00<br>0.44
202	2<br>66.67<br>1.68	1<br>33.33<br>0.94	3<br>100.00<br>1.33
203	0<br>0.00<br>0.00	2<br>100.00<br>1.89	2<br>100.00<br>0.89
204	0<br>0.00<br>0.00	1<br>100.00<br>0.94	1<br>100.00<br>0.44
205	0<br>0.00<br>0.00	1<br>100.00<br>0.94	1<br>100.00<br>0.44
207	1<br>100.00<br>0.84	0<br>0.00<br>0.00	1<br>100.00<br>0.44

216	1<br>100.00<br>0.84	0<br>0.00<br>0.00	1<br>100.00<br>0.44
233	0<br>0.00<br>0.00	1<br>100.00<br>0.94	1<br>100.00<br>0.44
240	0<br>0.00<br>0.00	1<br>100.00<br>0.94	1<br>100.00<br>0.44
242	1<br>100.00<br>0.84	0<br>0.00<br>0.00	1<br>100.00<br>0.44
296	2<br>100.00<br>1.68	0<br>0.00<br>0.00	2<br>100.00<br>0.89
297	1<br>100.00<br>0.84	0<br>0.00<br>0.00	1<br>100.00<br>0.44
300	0<br>0.00<br>0.00	1<br>100.00<br>0.94	1<br>100.00<br>0.44
303	0<br>0.00<br>0.00	1<br>100.00<br>0.94	1<br>100.00<br>0.44
315	0<br>0.00<br>0.00	1<br>100.00<br>0.94	1<br>100.00<br>0.44
316	2<br>28.57<br>1.68	5<br>71.43<br>4.72	7<br>100.00<br>3.11
318	0<br>0.00<br>0.00	1<br>100.00<br>0.94	1<br>100.00<br>0.44
320	1<br>50.00<br>0.84	1<br>50.00<br>0.94	2<br>100.00<br>0.89
331	0<br>0.00<br>0.00	1<br>100.00<br>0.94	1<br>100.00<br>0.44
395	2	3	5

	40.00	60.00	100.00
	1.68	2.83	2.22
397	0	1	1
	0.00	100.00	100.00
	0.00	0.94	0.44
401	2	0	2
	100.00	0.00	100.00
	1.68	0.00	0.89
403	2	0	2
	100.00	0.00	100.00
	1.68	0.00	0.89
414	1	0	1
	100.00	0.00	100.00
	0.84	0.00	0.44
423	0	1	1
	0.00	100.00	100.00
	0.00	0.94	0.44
450	1	0	1
	100.00	0.00	100.00
	0.84	0.00	0.44
477	0	1	1
	0.00	100.00	100.00
	0.00	0.94	0.44
521	1	0	1
	100.00	0.00	100.00
	0.84	0.00	0.44
548	0	1	1
	0.00	100.00	100.00
	0.00	0.94	0.44
551	1	0	1
	100.00	0.00	100.00
	0.84	0.00	0.44
557	2	0	2
	100.00	0.00	100.00
	1.68	0.00	0.89
562	2	0	2
	100.00	0.00	100.00
	1.68	0.00	0.89
565	1	1	2
	50.00	50.00	100.00

	0.84	0.94	0.89
566	1	1	2
	50.00	50.00	100.00
	0.84	0.94	0.89
568	0	1	1
	0.00	100.00	100.00
	0.00	0.94	0.44
570	0	1	1
	0.00	100.00	100.00
	0.00	0.94	0.44
572	1	0	1
	100.00	0.00	100.00
	0.84	0.00	0.44
576	2	6	8
	25.00	75.00	100.00
	1.68	5.66	3.56
Total	119	106	225
	52.89	47.11	100.00
	100.00	100.00	100.00

```
121 .
122 . local DISc LOS tariffa NUMmedDISCH

123 .
124 . foreach var of local DISc       {
  2.         di _newl "*`var' & CASE"
  3.         di _newl
  4.         tabstat `var', by(CASE) c(s)  s(n mea sd min q max) f(%4.2f)
  5.         //ttest `var', by(CASE)
125 .         ranksum `var', by(CASE)
  6.         di _newl
  7.         }

*LOS & CASE



Summary for variables: LOS
     by categories of: CASE (HolidayCase)
```

CASE	N	mean	sd	min	p25	p50	p75
regular	**121.00**	**15.17**	**15.02**	**3.00**	**9.00**	**11.00**	**17.00**
holiday	**106.00**	**22.25**	**38.16**	**3.00**	**11.00**	**15.50**	**23.00**
Total	**227.00**	**18.48**	**28.44**	**3.00**	**9.00**	**13.00**	**21.00**

CASE	max
regular	**155.00**
holiday	**390.00**
Total	**390.00**

Two-sample Wilcoxon rank-sum (Mann-Whitney) test

CASE	obs	rank sum	expected
regular	**121**	**12236**	**13794**
holiday	**106**	**13642**	**12084**
combined	**227**	**25878**	**25878**

```
unadjusted variance   243694.00
adjustment for ties     -597.65
                      ----------
adjusted variance     243096.35

Ho: LOS(CASE==regular) = LOS(CASE==holiday)
             z =  -3.160
    Prob > |z| =   0.0016
```

***tariffa & CASE**

Summary for variables: tariffa
     by categories of: CASE (HolidayCase)

CASE	N	mean	sd	min	p25	p50	p75
regular	**119.00**	**4290.29**	**2396.04**	**1090.00**	**3155.00**	**3642.00**	**4689.00**
holiday	**106.00**	**5195.97**	**3980.52**	**1072.00**	**3384.00**	**3983.50**	**5101.00**
Total	**225.00**	**4716.97**	**3264.46**	**1072.00**	**3333.00**	**3642.00**	**4986.00**

CASE	max
regular	**13658.00**
holiday	**23107.00**
Total	**23107.00**

Two-sample Wilcoxon rank-sum (Mann-Whitney) test

CASE	obs	rank sum	expected
regular	**119**	**12350.5**	**13447**
holiday	**106**	**13074.5**	**11978**
combined	**225**	**25425**	**25425**

```
unadjusted variance   237563.67
adjustment for ties     -968.20
                     ----------
adjusted variance     236595.47

Ho: tariffa(CASE==regular) = tariffa(CASE==holiday)
             z =  -2.254
    Prob > |z| =   0.0242
```

***NUMmedDISCH & CASE**

Summary for variables: NUMmedDISCH
     by categories of: CASE (HolidayCase)

CASE	N	mean	sd	min	p25	p50	p75
regular	**120.00**	**9.27**	**4.54**	**0.00**	**6.50**	**9.00**	**13.00**
holiday	**104.00**	**9.11**	**4.97**	**0.00**	**6.00**	**9.00**	**12.00**
Total	**224.00**	**9.19**	**4.74**	**0.00**	**6.00**	**9.00**	**12.00**

CASE	max
regular	**20.00**
holiday	**22.00**
Total	**22.00**

Two-sample Wilcoxon rank-sum (Mann-Whitney) test

```
        CASE |      obs    rank sum    expected
-------------+---------------------------------
     regular |      120     13620.5       13500
     holiday |      104     11579.5       11700
-------------+---------------------------------
    combined |      224       25200       25200

unadjusted variance   234000.00
adjustment for ties    -1226.21
                     ----------
adjusted variance     232773.79

Ho: NUMmed~H(CASE==regular) = NUMmed~H(CASE==holiday)
             z =   0.250
    Prob > |z| =   0.8028



126 .
127 . display in smcl "{newpage}"
```

```
128 .
129 . *OUTCOME
130 .
131 . local OUT DEADinhosp DEAD30 READMISS30 DEAD_READM30

132 .
133 . foreach var of local OUT        {
  2.          di _newl "*`var' & CASE (no test, description only"
  3.          di _newl
  4.          tab `var' CASE, r co ex
  5.          di _newl
  6.          }
```

*DEADinhosp & CASE (no test, description only

Key
frequency<br>*row percentage*<br>*column percentage*

DEADinhosp	HolidayCase regular	holiday	Total
0	**117**<br>**55.45**<br>**96.69**	**94**<br>**44.55**<br>**88.68**	**211**<br>**100.00**<br>**92.95**
1	**4**<br>**25.00**<br>**3.31**	**12**<br>**75.00**<br>**11.32**	**16**<br>**100.00**<br>**7.05**
Total	**121**<br>**53.30**<br>**100.00**	**106**<br>**46.70**<br>**100.00**	**227**<br>**100.00**<br>**100.00**

```
         Fisher's exact =                 0.021
 1-sided Fisher's exact =                 0.017
```

***DEAD30 & CASE (no test, description only**

Key
frequency
row percentage
column percentage

DEAD30	HolidayCase regular	holiday	Total
0	**115**	**92**	**207**
	**55.56**	**44.44**	**100.00**
	**95.04**	**86.79**	**91.19**
1	**6**	**14**	**20**
	**30.00**	**70.00**	**100.00**
	**4.96**	**13.21**	**8.81**
Total	**121**	**106**	**227**
	**53.30**	**46.70**	**100.00**
	**100.00**	**100.00**	**100.00**

Fisher's exact = **0.035**
1-sided Fisher's exact = **0.025**

***READMISS30 & CASE (no test, description only**

Key
frequency
row percentage
column percentage

READMISS30	HolidayCase regular	holiday	Total
0	**49**	**55**	**104**
	**47.12**	**52.88**	**100.00**
	**79.03**	**80.88**	**80.00**
1	**13**	**13**	**26**
	**50.00**	**50.00**	**100.00**
	**20.97**	**19.12**	**20.00**
Total	**62**	**68**	**130**
	**47.69**	**52.31**	**100.00**

	regular	holiday	Total
	100.00	100.00	100.00

Fisher's exact = 0.829
1-sided Fisher's exact = 0.482

***DEAD_READM30 & CASE (no test, description only**

Key
frequency
row percentage
column percentage

DEAD_READM 30	HolidayCase regular	holiday	Total
0	**104**	**82**	**186**
	**55.91**	**44.09**	**100.00**
	**85.95**	**77.36**	**81.94**
1	**17**	**24**	**41**
	**41.46**	**58.54**	**100.00**
	**14.05**	**22.64**	**18.06**
Total	**121**	**106**	**227**
	**53.30**	**46.70**	**100.00**
	**100.00**	**100.00**	**100.00**

Fisher's exact = **0.119**
1-sided Fisher's exact = **0.066**

```
134 .
135 . display in smcl "{newpage}"
```

```
136 .
137 .
138 . *---------------------------------------------
139 . *(Q2)INDEPENDENT CORRELATES OF HOLIDAY CASE
140 . *MULTIVARIABLE ANALYSIS
141 . *---------------------------------------------
142 .
143 . *check collinearity
144 .
145 . corr age sex edmonton_frail_scale_5 income_1000_month need_for_a_caregiver inappropri
    > ate_diet home_architect_barriers schooling_8_years
    (obs=226)

                 |      age      sex edmont~5 income~h need_f~r inappr~t home_a~s
    -------------+---------------------------------------------------------------
             age |   1.0000
             sex |   0.0644   1.0000
    edmonton_f~5 |   0.3412  -0.0307   1.0000
    income_100~h |  -0.0954   0.1354   0.0844   1.0000
    need_for_a~r |   0.3665   0.0955   0.5813   0.0654   1.0000
    inappropri~t |  -0.1552   0.1117   0.0490   0.1922   0.0142   1.0000
    home_archi~s |   0.0783   0.0432   0.0889   0.0101   0.1409   0.0585   1.0000
    schooling_~s |   0.4253   0.1516   0.2728   0.1637   0.2972  -0.0365   0.1282

                 | school~s
    -------------+---------
    schooling_~s |   1.0000


146 . //edmonton & need for a caregiver are collinear-choice is edmonton  (Marco)
147 .
148 . corr M_CCI_BIO M_CCI_SOC M_CCI_BEH M_CCI_ENV M_CCI_CUL
    (obs=237)

                 | M_CCI_~O M_CCI_~C M_CCI_~H M_CCI_~V M_CCI_~L
    -------------+---------------------------------------------
       M_CCI_BIO |   1.0000
       M_CCI_SOC |   0.0113   1.0000
       M_CCI_BEH |   0.0069   0.0785   1.0000
       M_CCI_ENV |   0.1950   0.1145   0.1252   1.0000
       M_CCI_CUL |   0.0184   0.1253   0.2494   0.1662   1.0000
```

```
149 .
150 . tab CASE
```

HolidayCase	Freq.	Percent	Cum.
regular	121	53.30	53.30
holiday	106	46.70	100.00
Total	227	100.00	

```
151 .
152 . *Model with single elements of CCI
153 .
154 . //with Edmonton frail scale
155 .
156 . logistic CASE age sex i.edmonton_frail_scale_5 i.income_1000_month  ///
>          i.inappropriate_diet i.home_architect_barriers i.schooling_8_years, nolog
```

```
Logistic regression                             Number of obs     =        226
                                                LR chi2(7)        =      27.13
                                                Prob > chi2       =     0.0003
Log likelihood = -142.52064                     Pseudo R2         =     0.0869
```

CASE	Odds Ratio	Std. Err.	z	P>|z|	[95% Conf.	Interval]
age	1.02	0.01	2.063	0.039	1.00	1.05
sex	1.07	0.31	0.231	0.817	0.60	1.89
edmonton_fr~5						
No	1.00	(base)				
Yes	1.85	0.66	1.732	0.083	0.92	3.72
income_1000~h						
No	1.00	(base)				
Yes	1.97	0.59	2.275	0.023	1.10	3.55
inappropria~t						
No	1.00	(base)				
Yes	2.29	0.90	2.101	0.036	1.06	4.95
home_archit~s						
No	1.00	(base)				
Yes	1.88	0.71	1.664	0.096	0.89	3.95
schooling_8~s						
No	1.00	(base)				
Yes	0.93	0.30	-0.230	0.818	0.49	1.75
_cons	0.04	0.04	-3.453	0.001	0.01	0.26

Note: **_cons** estimates baseline odds.

```
157 .
158 . estat gof
```

Logistic model for CASE, goodness-of-fit test

```
       number of observations =       226
 number of covariate patterns =       195
             Pearson chi2(187) =       186.25
                  Prob > chi2 =         0.5018
```

```
159 . lroc, nog
```

Logistic model for CASE

```
number of observations =      226
area under ROC curve   =   0.6900
```

```
160 .
161 . //with Need for caregiver
162 .
163 . logistic CASE age sex i.need_for_a_caregiver i.income_1000_month  ///
>         i.inappropriate_diet i.home_architect_barriers i.schooling_8_years, nolog
```

```
Logistic regression                               Number of obs     =        226
                                                  LR chi2(7)        =      26.00
                                                  Prob > chi2       =     0.0005
Log likelihood = -143.08456                       Pseudo R2         =     0.0833
```

CASE	Odds Ratio	Std. Err.	z	P>|z|	[95% Conf.	Interval]
age	1.03	0.01	2.143	0.032	1.00	1.05
sex	1.00	0.29	-0.016	0.987	0.56	1.76
need_for_a_~r						
No	1.00	(base)				
Yes	1.56	0.50	1.387	0.166	0.83	2.91
income_1000~h						
No	1.00	(base)				
Yes	2.01	0.60	2.335	0.020	1.12	3.61
inappropria~t						
No	1.00	(base)				
Yes	2.37	0.93	2.200	0.028	1.10	5.11
home_archit~s						
No	1.00	(base)				
Yes	1.83	0.69	1.584	0.113	0.87	3.85
schooling_8~s						
No	1.00	(base)				

Yes	**0.94**	**0.30**	**-0.198**	**0.843**	**0.50**	**1.77**
_cons	**0.05**	**0.05**	**-3.316**	**0.001**	**0.01**	**0.30**

Note: **_cons** estimates baseline odds.

```
164 .
165 . estat gof
```

Logistic model for CASE, goodness-of-fit test

```
       number of observations =       226
 number of covariate patterns =       197
            Pearson chi2(189) =       190.75
                  Prob > chi2 =         0.4506
```

```
166 . lroc, nog
```

Logistic model for CASE

```
number of observations =      226
area under ROC curve   =   0.6869
```

```
167 .
168 .
169 . *Model with CCI_TOT
170 .
171 . logistic CASE age sex CCI_TOT, nolog
```

```
Logistic regression                             Number of obs     =        227
                                                LR chi2(3)        =      13.31
                                                Prob > chi2       =     0.0040
Log likelihood = -150.19354                     Pseudo R2         =     0.0424
```

CASE	Odds Ratio	Std. Err.	z	P>|z|	[95% Conf.	Interval]
age	**1.02**	**0.01**	**1.655**	**0.098**	**1.00**	**1.04**
sex	**1.17**	**0.32**	**0.563**	**0.573**	**0.68**	**2.00**
CCI_TOT	**1.06**	**0.03**	**2.466**	**0.014**	**1.01**	**1.12**
_cons	**0.08**	**0.07**	**-3.114**	**0.002**	**0.02**	**0.39**

Note: **_cons** estimates baseline odds.

```
172 .
173 .
174 . *Model with components of CCI
175 .
176 . logistic CASE age sex i.M_CCI_BIO i.M_CCI_SOC ///
>         i.M_CCI_BEH i.M_CCI_ENV i.M_CCI_CUL, nolog
```

```
Logistic regression                             Number of obs     =        227
                                                LR chi2(7)        =      15.04
                                                Prob > chi2       =     0.0355
Log likelihood = -149.32987                     Pseudo R2         =     0.0479
```

CASE	Odds Ratio	Std. Err.	z	P>|z|	[95% Conf.	Interval]
age	**1.03**	**0.01**	**2.505**	**0.012**	**1.01**	**1.05**
sex	**1.24**	**0.35**	**0.764**	**0.445**	**0.72**	**2.15**
M_CCI_BIO						
1	**1.00**	(base)				
2	**1.07**	**0.33**	**0.219**	**0.827**	**0.58**	**1.97**
M_CCI_SOC						
1	**1.00**	(base)				
2	**1.63**	**0.66**	**1.218**	**0.223**	**0.74**	**3.59**
M_CCI_BEH						
1	**1.00**	(base)				
2	**1.93**	**0.65**	**1.951**	**0.051**	**1.00**	**3.74**
M_CCI_ENV						
1	**1.00**	(base)				
2	**1.32**	**0.38**	**0.973**	**0.331**	**0.75**	**2.31**
M_CCI_CUL						
1	**1.00**	(base)				
2	**1.02**	**0.31**	**0.079**	**0.937**	**0.57**	**1.85**
_cons	**0.06**	**0.06**	**-3.153**	**0.002**	**0.01**	**0.35**

Note: **_cons** estimates baseline odds.

```
177 .
178 . estat gof

    Logistic model for CASE, goodness-of-fit test
    ---------------------------------------------

           number of observations =       227
     number of covariate patterns =       202
                Pearson chi2(194) =       204.59
                      Prob > chi2 =         0.2871

179 . lroc, nog

    Logistic model for CASE

    number of observations =      227
    area under ROC curve   =   0.6306

180 .
181 .
182 . display in smcl "{newpage}"
```
